# Supplementary material for: Identification and prevalence of frailty in diabetes mellitus and association with clinical outcomes: a systematic review protocol
Source: BMJ Open. 2020 Sep 1;10(9):e037476. doi: 10.1136/bmjopen-2020-037476 (PMC7467518; doi:10.1136/bmjopen-2020-037476)
Supplement: Supplementary data [file bmjopen-2020-037476supp001.pdf]

Medline Search Strategy

Search Terms

1. Exp Frailty/
2. Exp Frail Elderly/
3. Frail*.tw
4. 1 or 2 or 3
5. Exp Diabetes Mellitus
6. Diabet*.tw
7. (IDDM or NIDDM or MODY or T1DM, or T2DM or T1D or T2D).tw
8. (non insulin* depend* or non insulin depend* or non insulin?depend* or non insulin ?depend).tw
9. (insulin* depend* or insulin ?depend*).tw
10. 5 or 6 or 7 or 8 or 9
11. Exp Diabetes Insipidus/
12. Diabet* insipidus.tw
13. 11 or 12
14. 10 not 13
15. 4 and 14

Language restriction

None applied to search (non-English language studies excluded at screening stage)

Years searched

2001-November 2019
