# Supplementary material for: Identification and prevalence of frailty in diabetes mellitus and association with clinical outcomes: a systematic review protocol
Source: BMJ Open. 2020 Sep 1;10(9):e037476. doi: 10.1136/bmjopen-2020-037476 (PMC7467518; doi:10.1136/bmjopen-2020-037476)
Supplement: Supplementary data [file bmjopen-2020-037476supp002.pdf]

The Newcastle-Ottawa Scale

Adaptation for studies assessing the prevalence and impact of frailty in diabetes

1 – Representativeness of the exposed (i.e. frail) cohort

a) Truly representative (one star)

b) Somewhat representative (one star)

c) Selected group

d) No description of the derivation of the cohort

2 – Selection of the non-exposed (i.e. non-frail) cohort

a) Drawn from the same community as the exposed cohort (one star)

b) Drawn from a different source

c) No description of the derivation of the non-exposed cohort

3 – Ascertainment of exposure

a) Validated measurement tool for frailty (two stars)

b) Non-validated measurement tool, but the tool is available or described (one star)

c) No description of measurement tool

4 – Non-respondents

a) Comparability between respondents and non-respondents' characteristics is established, and the response rate is satisfactory (one star)
b) The response rate is unsatisfactory, or the comparability between respondents and non-respondents is unsatisfactory

c) No description of the response rate of the characteristics of the responders and non-responders

5 – Demonstration that outcome of interest was not present at the start of the study

a) Yes (one star)

b) No

Comparability:

1 – Comparability of the cohorts on the basis of the design or analysis being controlled for confounders

a) The study controls for age and sex (one star)

b) The study controls for other factors (one star)

c) Cohorts are not comparable on the basis of the design or analysis controlled for confounders

Outcomes:

1 – Assessment of outcomes

a) Independent assessment (one star)

b) Record linkage (one star)

c) Self-report

d) No description

e) Other

2 – Follow-up long enough for outcomes to occur

a) Yes (one star)

b) No

3 – Adequacy of follow-up of cohorts

a) Complete follow-up: all subjects accounted for (one star)

b) Subjects lost to follow-up unlikely to introduce bias – number lost less than or equal to 20% or description of those lost suggested no different from those followed (one star)

c) Follow-up rate less than 80% and no description of those lost

d) No statement
