# Supplementary material for: Identification and prevalence of frailty in diabetes mellitus and association with clinical outcomes: a systematic review protocol
Source: BMJ Open. 2020 Sep 1;10(9):e037476. doi: 10.1136/bmjopen-2020-037476 (PMC7467518; doi:10.1136/bmjopen-2020-037476)
Supplement: Author's manuscript [file bmjopen-2020-037476.draft_revisions.pdf]

BMJ Open

BMJ Open is committed to open peer review. As part of this commitment we make the peer review history of every article we publish publicly available.

When an article is published we post the peer reviewers' comments and the authors' responses online. We also post the versions of the paper that were used during peer review. These are the versions that the peer review comments apply to.

The versions of the paper that follow are the versions that were submitted during the peer review process. They are not the versions of record or the final published versions. They should not be cited or distributed as the published version of this manuscript.

BMJ Open is an open access journal and the full, final, typeset and author-corrected version of record of the manuscript is available on our site with no access controls, subscription charges or pay-per-view fees (http://bmjopen.bmj.com).

If you have any questions on BMJ Open's open peer review process please email info.bmjopen@bmj.com

BMJ Open

The prevalence of frailty in diabetes mellitus and association with clinical outcomes: A systematic review protocol

Journal:	*BMJ Open*
Manuscript ID	bmjopen-2020-037476
Article Type:	Protocol
Date Submitted by the Author:	04-Feb-2020
Complete List of Authors:	Hanlon, Peter; University of Glasgow Institute of Health and Wellbeing, ;<br><br>Fauré, Isabella ; University of Glasgow Institute of Health and Wellbeing<br>Corcoran, Neave ; University of Glasgow Institute of Health and Wellbeing<br>Butterly, Elaine; University of Glasgow Institute of Health and Wellbeing<br>McAllister, David; University of Glasgow Institute of Health and Wellbeing<br>Mair, Frances; University of Glasgow Institute of Health and Wellbeing
Keywords:	Diabetes & endocrinology < INTERNAL MEDICINE, EPIDEMIOLOGY, GERIATRIC MEDICINE

SCHOLARONE™
Manuscripts

I, the Submitting Author has the right to grant and does grant on behalf of all authors of the Work (as defined in the below author licence), an exclusive licence and/or a non-exclusive licence for contributions from authors who are: i) UK Crown employees; ii) where BMJ has agreed a CC-BY licence shall apply, and/or iii) in accordance with the terms applicable for US Federal Government officers or employees acting as part of their official duties; on a worldwide, perpetual, irrevocable, royalty-free basis to BMJ Publishing Group Ltd ("BMJ") its licensees and where the relevant Journal is co-owned by BMJ to the co-owners of the Journal, to publish the Work in this journal and any other BMJ products and to exploit all rights, as set out in our licence.

The Submitting Author accepts and understands that any supply made under these terms is made by BMJ to the Submitting Author unless you are acting as an employee on behalf of your employer or a postgraduate student of an affiliated institution which is paying any applicable article publishing charge ("APC") for Open Access articles. Where the Submitting Author wishes to make the Work available on an Open Access basis (and intends to pay the relevant APC), the terms of reuse of such Open Access shall be governed by a Creative Commons licence – details of these licences and which Creative Commons licence will apply to this Work are set out in our licence referred to above.

Other than as permitted in any relevant BMJ Author's Self Archiving Policies, I confirm this Work has not been accepted for publication elsewhere, is not being considered for publication elsewhere and does not duplicate material already published. I confirm all authors consent to publication of this Work and authorise the granting of this licence.

For peer review only

The prevalence of frailty in diabetes mellitus and association with clinical outcomes: A systematic review protocol

Authors:

Dr Peter Hanlon[1]
Ms Isabella Fauré[1]
Dr Neave Corcoran[1]
Dr Elaine Butterly[2]
Dr David McAllister[2]
Professor Frances S Mair[1]

Affiliations:

1. General Practice and Primary Care, Institute of Health and Wellbeing, University of Glasgow, Glasgow, UK
2. Public Health, Institute of Health and Wellbeing, University of Glasgow, Glasgow, UK

Corresponding author:

Dr Peter Hanlon
General Practice and Primary Care
Institute of Health and Wellbeing
University of Glasgow
1 Horselethill Road
Glasgow, G12 9LX

Peter.hanlon@glasgow.ac.uk
+44 141 330 8383

For peer review only

For peer review only - http://bmjopen.bmj.com/site/about/guidelines.xhtml

Word count: 2311

Keywords: Frailty, Diabetes Mellitus, Systematic review, Protocol

For peer review only

For peer review only - http://bmjopen.bmj.com/site/about/guidelines.xhtml

Abstract

Introduction:

Diabetes mellitus is common and growing in prevalence, and an increasing proportion of people with diabetes are living to older age. Frailty is therefore becoming an important concept in diabetes. Frailty is associated with older age and describes a state of increased susceptibility to decompensation in response to physiological stress. A range of measures have been used to quantify frailty. This systematic review aims to identify measures used to quantify frailty in people with diabetes (type 1, type 2, or unspecified); to summarize the prevalence of frailty in diabetes; and to describe the relationship between frailty and adverse clinical outcomes in people with diabetes.

Methods and analysis:

Three electronic databases (Medline, Embase and Web of Science) will be searched from 2000 to November 2019 and supplemented by citation searching of relevant articles and hand-searching of reference lists. Two reviewers will independently review titles, abstracts and full texts. Inclusion criteria include: (1) Adults with diabetes mellitus (type 1, type 2, or unspecified); (2) Quantify frailty using any validated frailty measure; (3) Report the prevalence of frailty and/or the association between frailty and clinical outcomes in people with diabetes; (4) Studies that assess generic (e.g. mortality, hospital admission, falls) or diabetes specific outcomes (e.g. hypoglycaemic episodes, cardiovascular events, diabetic nephropathy, diabetic retinopathy); (5) Cross-sectional and longitudinal observational studies. Study quality will be assessed using the Newcastle-Ottawa scale for observational studies. Clinical and methodological heterogeneity will be assessed, and a random effects meta-analysis performed if appropriate. Otherwise, a narrative synthesis will be performed.

For peer review only

Ethics and dissemination:

This study will summarise current knowledge about measurement, prevalence and implications of frailty in diabetes. This will inform future research and clinical guidelines to assess the balance of risks and treatment priorities in the growing number of people living with frailty and diabetes.

Registration number:

PROSPERO CRD42020163109.

Strengths and Limitations

This systematic review will provide a comprehensive overview of the prevalence and implications of frailty in people with diabetes.

We will include a broad range of frailty definitions and clinical outcomes relevant to diabetes.

There is likely to be significant heterogeneity between population characteristics and frailty definitions in included studies.

For peer review only

For peer review only - http://bmjopen.bmj.com/site/about/guidelines.xhtml

Introduction

The prevalence of diabetes mellitus is increasing across the world. Population demographics are also shifting towards an ageing population. Among people above the age of 65, the prevalence of diabetes can be as high as 30%.(1) Diabetes in older people is therefore a growing clinical and public health priority. One factor with important implications for disease management in older age is frailty.

Frailty is a state characterised by reduced functional reserve across multiple physiological systems.(2) People living with frailty have impaired resolution of homeostasis following physiological stressors. Frailty therefore carries an increased risk of a range of adverse health outcomes, such as falls, cognitive decline, hospital admission and mortality.(3) Frailty is widely recognised to be a multidimentional and dynamic state, associated with older age and with a range of non-communicable diseases.(3) However, there is no single universally accepted operational definition of frailty. Rather, a wide range of definitions have been utilized in both research and clinical practice.(4)

The two dominant paradigms in the frailty literature are the frailty phenotype and the frailty index. The frailty phenotype, described by *Fried et al* in 2001, defines frailty as the presence of three or more out of five features: low hand-grip strength, unintentional weight loss, low physical activity, exhaustion, and slow walking pace.(5) The presence of one or two of these features is classified as a pre-frail state. The frailty index, described by Rockwood and Mitnitski in 2007, is based on a cumulative-deficit model of frailty whereby frailty is identified by counting the number of health 'deficits' present in an individual.(6) At least 30 deficits are required to construct a frailty index, all of which must increase in prevalence with age, be associated with poor health, and not saturate too early (i.e. be universally present among older people).(7) Both the frailty phenotype and frailty index have been associated with adverse health outcomes in a range of older populations, however the populations identified as frail by each are different. Since their original description, a wide range of

For peer review only - http://bmjopen.bmj.com/site/about/guidelines.xhtml

For peer review only

other frailty instruments, as well as adaptations of the frailty index and phenotype, have been developed for both epidemiological studies and for clinical practice.(3, 4)

The relationship between diabetes mellitus and frailty is complex. Diabetes is associated with a higher prevalence of frailty.(8-11) Both type 1 and type 2 diabetes lead to microvascular and macrovascular complications which have important physical, cognitive and functional consequences, that may contribute to the development of frailty. Hyperglycaemia is also recognized to directly impact muscle mass and quality, exacerbating age-related sarcopenia and, in turn, physical function.(12) However, the association between frailty and poor functional outcomes in people with diabetes is only partially explained by direct complications of diabetes.(10, 13)

The importance of frailty in the context of diabetes is increasingly recognised in clinical guidelines. Specifically, higher HbA1c targets are recommended in the context of frailty, in part due to the increased risks associated with hypoglycaemia.(14) Despite this, up to 40% of older people with diabetes may be over-treated (with HbA1c <7%).(15, 16) Conversely, poor glycaemic control and associated vascular complications risk causing, or accelerating the progression of, frailty.(17)

One recent meta-analysis demonstrated a consistent relationship between frailty and mortality, hospitalisation, and cardiovascular events in the context of diabetes.(18) We are not aware of any systematic review to assess the prevalence of frailty in diabetes, or to consider a broader range of outcomes relevant to the management of diabetes. Given the risks of both over- and under-treatment of diabetes in the context of frailty, understanding the range of potential associations is required to inform clinical decisions and to underpin future research.

To enhance understanding of the implications and management of diabetes within an ageing population, it is important to fully describe the association between diabetes and frailty. Given the risks of both over- and under-treatment of diabetes in the context of frailty, it is important to understand the associations between frailty and a range of potential outcomes in diabetes. This includes generic outcomes such as mortality and hospitalisation and disability and disease specific

For peer review only

For peer review only - http://bmjopen.bmj.com/site/about/guidelines.xhtml

outcomes such as retinopathy, neuropathy, and hypoglycaemic events. An understanding of the range and complexity of these associations is required to inform clinical decisions around treatment priorities and to underpin future research. This includes quantifying the prevalence of frailty in people with diabetes, and the impact that different frailty definitions might have on this prevalence. This manuscript describes the protocol of a systematic review aiming to synthesise existing evidence relating to these questions.

Aims

The systematic review will aim to:

- Identify which frailty measures have been used to assess frailty in people with diabetes mellitus (type1, type 2, or mixed/unspecified)
- Quantify the prevalence of frailty among people with diabetes
- Describe the association between frailty and both generic (e.g. mortality) and disease specific (e.g. hypoglycaemia) clinical outcomes in the context of diabetes

For peer review only

Methods and analysis

This protocol is registered with PROSPERO (CRD42020163109). The review will be conducted and reported according to the Preferred Reporting Items for Systematic Reviews and Meta-Analyses (PRISMA) statement.(19)

Eligibility criteria for inclusion

The eligibility criteria for this review are summarized in table 1 and explained in more detail below.

Table 1. Inclusion Criteria

PICOS component	Description
Population	Adults (≥ 18 years old)<br><br>Diabetes mellitus (type 1, type 2, or unspecified)
Exposure	Frailty as assessed by a validated frailty measure
Comparator	People with diabetes not classified as frail
Outcomes	Generic:<br>• Mortality<br>• Major Adverse Cardiovascular Events<br>• Hospital admission<br>• Admission to long-term care facility<br>• Falls<br>• Number of clinic attendances<br>• Quality of life<br>• Disability/functional status<br>Diabetes specific:<br>• HbA1c (cross sectional association, or longitudinal)

For peer review only - http://bmjopen.bmj.com/site/about/guidelines.xhtml

	• Glycaemic variability<br>• Hypoglycaemic episodes<br>• Diabetic retinopathy (cross sectional association, or longitudinal)<br>• Diabetic nephropathy (cross sectional association, or longitudinal)<br>○ Include development of end-stage renal disease<br>• Diabetic foot complications (cross sectional association, or longitudinal)<br>• Treatment burden (e.g. Diabetic Treatment Burden Questionnaire)
Settings	Community (including care home/nursing home)<br>Outpatient clinic<br>Inpatient
Study design	Cross sectional or longitudinal<br>Cohort
Other exclusions	Conference abstracts, letters, review articles, intervention studies

Population

We will include studies analysing data from people with any form of diabetes mellitus.

From an initial scoping of the literature, it is likely that many studies describing frailty in older populations measure unspecified ‘diabetes’ rather than explicitly type 1 or type 2 diabetes. We will therefore include any study which includes people with type 1, type 2 diabetes, or people with unspecified diabetes. Given that frailty is a state associated with older age, and that type 2 diabetes is both more prevalent than type 1 diabetes and becomes more prevalent with age, it is likely that most (but not all) people with diabetes in the relevant populations will have type 2 diabetes. Studies

For peer review only

For peer review only - http://bmjopen.bmj.com/site/about/guidelines.xhtml

of type 1 diabetes, type 2 diabetes and those of unspecified diabetes will be considered separately in any subsequent analysis.

We will include studies focusing purely on people with diabetes, or population-based studies that report results for people with diabetes separately.

Exposure

The 'exposure' of interest is frailty. Many epidemiological measures and clinical tools have been developed to identify frailty for research or clinical practice.(4)

To be eligible for inclusion, a study must use a measure which explicitly seeks to quantify frailty. We will include measures developed primarily as epidemiological tools (e.g. the frailty phenotype frailty index).(5, 6) We will also include measures designed primarily for clinical practice (e.g. the Clinical Frailty Scale).(20)

Studies focusing solely on comorbidity (i.e. no additional measures to identify 'frailty') will be excluded unless these are explicitly operationalised as a 'frailty index'. In this case studies would generally be expected to include additional deficits (such as symptoms, functional limitations, laboratory measures etc.). Studies which use a single parameter as a proxy for frailty (e.g. grip strength alone, self-rated health) will be excluded.

Comparator

Studies that report the prevalence of frailty will be eligible for inclusion if they report the prevalence of frailty in diabetes only. Studies should report the number or proportion of participants with and without frailty (or with varying degrees of frailty, depending on the measure used).

For assessing the association between frailty and clinical outcomes in the context of diabetes, studies should report the association with the outcome in the presence of absence of frailty (if a binary or categorical measure is used) or by degree of frailty.

For peer review only - http://bmjopen.bmj.com/site/about/guidelines.xhtml

Outcomes

Outcomes of interest are summarized in table 1. We will include studies assessing any of these outcomes as long as the association is specifically quantified in people with diabetes and frailty.

Setting

We will include studies of community-dwelling patients, outpatient populations or hospital inpatients.

For the purposes of this review, given the focus on frailty, people living in long-term care facilities (e.g. care-homes, nursing-homes) will be considered to be 'community-dwelling'. Therefore, any study including, or specifically recruiting, nursing home residents will be eligible for inclusion.

Identification of studies

Electronic searches

Medline, Embase, and Web of Science (Core collection) databases will be search using a combination of Medical Subject Headings (MeSH) and keyword searches. The terms used for the medline search are shown in table 2. These terms will be adapted for the other databases. Searches will be from 2000 to November 2019. The year 2000 was chosen as the start date as the first seminal paper operationalising the concept of frailty in an epidemiological study was published in 2001. Articles published prior to this date are therefore unlikely to be relevant. No language restriction will be applied to the search, but only English language articles will be included at the screening level.

Table 2: Medline Search
1. Exp Frailty/<br>2. Exp Frail Elderly/

For peer review only - http://bmjopen.bmj.com/site/about/guidelines.xhtml

3. Frail*.tw
4. 1 or 2 or 3
5. Exp Diabetes Mellitus
6. Diabet*.tw
7. (IDDM or NIDDM or MODY or T1DM, or T2DM or T1D or T2D).tw
8. (non insulin* depend* or non insulin depend* or non insulin?depend* or non insulin ?depend).tw
9. (insulin* depend* or insulin ?depend*).tw
10. 5 or 6 or 7 or 8 or 9
11. Exp Diabetes Insipidus/
12. Diabet* insipidus.tw
13. 11 or 12
14. 10 not 13
15. 4 and 14

178 ## Identifying additional articles

179 Electronic searches will be supplemented by hand searching reference lists of relevant articles. A
180 citation search of all relevant articles will also be carried out using the Web of Science citation search
181 tool.

For peer review only

For peer review only - http://bmjopen.bmj.com/site/about/guidelines.xhtml

Data collection and analysis

Selection of studies

Two reviewers, working independently, will screen all titles and abstracts of records identified in the database searches. PICOS criteria outlined above will be used to determine eligibility. Where there is disagreement, studies will be retained for full-text screening.

Full texts of all potentially eligible studies will be screened independently by two reviewers. Disagreements about eligibility will be resolved by consensus, involving a third reviewer where necessary.

Data extraction

A standard data extraction form will be designed and piloted before being applied to each of the included studies. Extracted data will include:

Study details

- Author
- Year
- Location
- Setting (community, outpatient, residential care)
- Method of recruitment (e.g. random sample, postal invitation, consecutive patients)
- Method of assessment (face-to-face, survey, linkage to healthcare records)

Population

- Age
- Sex
- Ethnicity

For peer review only

For peer review only - http://bmjopen.bmj.com/site/about/guidelines.xhtml

- Socioeconomic status
- Comorbidities
- Medications
- Social circumstances (e.g. living independently, requiring carers, family support etc)
- Smoking status
- Physical activity

Diabetes details

- Type of diabetes (type 1, type 2 or unspecified)
- Method of confirmation (self-report, medical records, clinical assessment)
- Measure of control (e.g. HbA1c)
- Medication (e.g. proportion taking insulin, oral antidiabetics etc.)
- Presence and severity of complications (e.g. retinopathy, nephropathy, neuropathy, ulceration, Charcot arthropathy)

Frailty definition

- Frailty measure used
- Definitions for each component of the frailty measure (e.g. cut-points used for continuous measures, method of assessment (questionnaire, interview etc.))

Frailty prevalence

Outcomes (generic):

- Mortality
- Major Adverse Cardiovascular Events
- Hospital admission
- Admission to long-term care facility
- Falls

For peer review only

For peer review only - http://bmjopen.bmj.com/site/about/guidelines.xhtml

- Number of clinic attendances
- Quality of life
- Disability/functional status

Outcomes (diabetes specific):

- HbA1c (cross sectional association, or longitudinal)
- Glycaemic variability
- Hypoglycaemic episodes
- Diabetic retinopathy (cross sectional association, or longitudinal)
- Diabetic nephropathy (cross sectional association, or longitudinal)
- Diabetic foot complications (cross sectional association, or longitudinal)
- Treatment burden (e.g. Diabetic Treatment Burden Questionnaire)

For each outcome reported we will record

- The association between frailty and the outcome (e.g. prevalence, odds ratio, hazard ratio etc.)
- Adjustment for any potential confounders
- Length of follow-up over which the outcome was assessed
- Method of analysis of competing risks when assessing each outcome

Assessment of methodological quality

The Newcastle-Ottawa scale will be used to assess the risk of bias for each study.(21) This scale is widely used for the assessment of observational studies. Where studies are purely cross-sectional, an adapted version of the Newcastle-Ottawa scale will be applied to assess risk of bias in selection, comparability, and exposure. In assessing the comparability of frail/non-frail groups, age will be taken as the most important factor for which studies should account.

For peer review only

For peer review only - http://bmjopen.bmj.com/site/about/guidelines.xhtml

Data synthesis

The appropriate method of data synthesis will be determined after assessment of the heterogeneity of the included studies, in terms of population selection and demographics, frailty definition, and method of outcome assessment.

If appropriate, we will combine these in a random effects meta-analysis (anticipating heterogeneity in the true association). As well as a pooled estimate and 95% confidence intervals, we will also calculate the prediction interval to assess the range of plausible estimates from the observed data. Heterogeneity will be quantified using the I^2 statistic. Where heterogeneity is present, we will attempt to explore potential sources of heterogeneity using subgroup analyses (e.g. by method of determining frailty, age of sample population, method of outcome assessment). By doing so, we propose to explore factors that may influence the estimates reported in observational studies in the presence of heterogeneity, rather than provide a definitive single estimate.(22)

Only those studies that are judged to be sufficiently comparable will be included in meta-analyses. For outcomes where there are too few studies, or the included studies are too heterogenous to permit a meaningful meta-analysis (for example, in terms of outcome definition or method of assessing frailty), we will perform a narrative synthesis of the study findings. This will report the methods used to identify frailty along with the prevalence and association with outcomes, to explore the impact of the method of assessment on the observed relationship. This will be reported alongside detail of the recruitment strategy, age profile, and characteristics of each sample included.

For peer review only - http://bmjopen.bmj.com/site/about/guidelines.xhtml

Ethics and dissemination

This systematic review will provide an overview of the prevalence of frailty in diabetes, and the relationship between frailty and adverse health outcomes in people with diabetes.

As the prevalence of both frailty and diabetes increase, it will become increasingly important for clinical guidelines for the treatment of diabetes to explicitly consider the needs of people living with frailty. Quantifying the prevalence of frailty in diabetes will allow the scale of this challenge to be better appreciated. By including any reported definition of frailty within our inclusion criteria, this review will demonstrate which of the wide range of frailty instruments and measures have been used to study frailty in diabetes. It will also be possible to compare if and how prevalence and association with outcomes differs depending on the frailty definition used.

Given the likely heterogeneity in frailty definitions, as well as inherent differences in the populations studied, it may not be possible to undertake a meta-analysis of the findings of this review. If this is the case, we propose to conduct a detailed narrative synthesis, systematically describing and synthesizing details of the populations under study as well as the details of frailty definitions used.

We also propose to search for and extract data for a wide range of clinical outcomes. Given the multidimentional nature of frailty, and the vulnerability to decompensation that is inherent to any frailty definition, it is likely that frailty will be associated with a range of adverse outcomes. The challenge in translating these associations into meaningful recommendations is understanding the balance of these risks, and how they might inform clinical decisions and recommendations. The balance of risks in diabetes, and treatment priorities, may differ depending on the degree of frailty experienced by an individual. The associations may also differ in their nature or magnitude depending on the method used to identify frailty. This review will aim to provide an overview of what is known about the relationship between frailty and both generic and disease specific

For peer review only - http://bmjopen.bmj.com/site/about/guidelines.xhtml

outcomes. This is likely to inform priorities for future research into the consequences of frailty in diabetes.

As this project is a systematic review, ethical approval is not required. Patients or the public were not involved in the development of this protocol.

For peer review only

Author contributions

All authors (PH, IF, NC, DM and FM) contributed to the conception and design of the proposed study. PH, DM and FM developed the data sources and search strategy. PH, IF, NC, DM and FM refined the inclusion criteria. PH, IF, NC, DM and FM developed the data extraction template which was piloted by PH, IF and NC. PH and IF wrote the first draft. All authors critically reviewed this and subsequent drafts. All authors approved the final version of the manuscript for submission. FM is the guarantor of the review. All authors accept accountability for the accuracy of the protocol.

Funding statement

Dr Peter Hanlon was funded by a Medical Research Council Clinical Research Training Fellowship (Grant reference MR/S021949/1) entitled "Understanding prevalence and impact of frailty in chronic disease and implications for clinical management. The funder had no role in protocol development.

Competing interests statement

The authors declare no competing interests.

For peer review only

For peer review only - http://bmjopen.bmj.com/site/about/guidelines.xhtml

References

1. Sinclair AJ, Rodriguez-Mañas L. Diabetes and Frailty: Two Converging Conditions? Canadian Journal of Diabetes. 2016;40(1):77-83.
2. Clegg A, Young J, Iliffe S, Rikkert MO, Rockwood K. Frailty in elderly people. The Lancet. 2013;381(9868):752-62.
3. Hoogendijk EO, Afilalo J, Ensrud KE, Kowal P, Onder G, Fried LP. Frailty: implications for clinical practice and public health. The Lancet. 2019;394(10206):1365-75.
4. Bouillon K, Kivimaki M, Hamer M, Sabia S, Fransson EI, Singh-Manoux A, et al. Measures of frailty in population-based studies: an overview. BMC Geriatrics. 2013;13(1):64.
5. Fried LP, Tangen CM, Walston J, Newman AB, Hirsch C, Gottdiener J, et al. Frailty in Older Adults: Evidence for a Phenotype. The Journals of Gerontology: Series A. 2001;56(3):M146-M57.
6. Rockwood K, Mitnitski A. Frailty in Relation to the Accumulation of Deficits. The Journals of Gerontology: Series A. 2007;62(7):722-7.
7. Searle SD, Mitnitski A, Gahbauer EA, Gill TM, Rockwood K. A standard procedure for creating a frailty index. BMC Geriatrics. 2008;8(1):24.
8. Kotsani M, Chatziadamidou T, Economides D, Benetos A. Higher prevalence and earlier appearance of geriatric phenotypes in old adults with type 2 diabetes mellitus. Diabetes Research and Clinical Practice. 2018;135:206-17.
9. Thein FS, Li Y, Nyunt MSZ, Gao Q, Wee SL, Ng TP. Physical frailty and cognitive impairment is associated with diabetes and adversely impact functional status and mortality. Postgraduate Medicine. 2018;130(6):561-7.
10. Castro-Rodríguez M, Carnicero JA, Garcia-Garcia FJ, Walter S, Morley JE, Rodríguez-Artalejo F, et al. Frailty as a Major Factor in the Increased Risk of Death and Disability in Older People With Diabetes. Journal of the American Medical Directors Association. 2016;17(10):949-55.
11. Hanlon P, Hannigan L, Fischbacker C, Welton N, Dias S, Mair F, et al. Representation of people with comorbidity and multimorbidity in clinical trials of novel drug therapies; an individual-level participant data analysis. BMC Medicine (in press). 2019.
12. Yoon JW, Ha Y-C, Kim KM, Moon JH, Choi SH, Lim S, et al. Hyperglycemia Is Associated with Impaired Muscle Quality in Older Men with Diabetes: The Korean Longitudinal Study on Health and Aging. Diabetes Metab J. 2016;40(2):140-6.
13. Maggi S, Noale M, Gallina P, Marzari C, Bianchi D, Limongi F, et al. Physical disability among older Italians with diabetes. The ILSA Study. Diabetologia. 2004;47(11):1957-62.
14. Schernthaner G, Schernthaner-Reiter MH. Diabetes in the older patient: heterogeneity requires individualisation of therapeutic strategies. Diabetologia. 2018;61(7):1503-16.
15. Formiga F, Franch-Nadal J, Rodriguez L, Ávila L, Fuster E. Inadequate glycaemic control and therapeutic management of adults over 65 years old with type 2 diabetes mellitus in Spain. The journal of nutrition, health & aging. 2017;21(10):1365-70.
16. Braun AK, Kubiak T, Kuntsche J, Meier-Höfig M, Müller UA, Feucht I, et al. SGS: a structured treatment and teaching programme for older patients with diabetes mellitus—a prospective randomised controlled multi-centre trial. Age and ageing. 2009;38(4):390-6.
17. Quartuccio M, Buta B, Kalyani RR. Comparative effectiveness for glycemic control in older adults with diabetes. Current geriatrics reports. 2017;6(3):175-86.
18. Ida S, Kaneko R, Imataka K, Murata K. Relationship between frailty and mortality, hospitalization, and cardiovascular diseases in diabetes: a systematic review and meta-analysis. Cardiovascular Diabetology. 2019;18(1):81.
19. Moher D, Liberati A, Tetzlaff J, Altman DG. Preferred reporting items for systematic reviews and meta-analyses: the PRISMA statement. BMJ. 2009;339:b2535.

For peer review only - http://bmjopen.bmj.com/site/about/guidelines.xhtml

20. Rockwood K, Song X, MacKnight C, Bergman H, Hogan DB, McDowell I, et al. A global clinical measure of fitness and frailty in elderly people. Canadian Medical Association Journal. 2005;173(5):489.

21. Stang A. Critical evaluation of the Newcastle-Ottawa scale for the assessment of the quality of nonrandomized studies in meta-analyses. European Journal of Epidemiology. 2010;25(9):603-5.

22. Mueller M, D'Addario M, Egger M, Cevallos M, Dekkers O, Mugglin C, et al. Methods to systematically review and meta-analyse observational studies: a systematic scoping review of recommendations. BMC Medical Research Methodology. 2018;18(1):44.

For peer review only

For peer review only - http://bmjopen.bmj.com/site/about/guidelines.xhtml

Reporting checklist for protocol of a systematic review.

Based on the PRISMA-P guidelines.

Instructions to authors

Complete this checklist by entering the page numbers from your manuscript where readers will find each of the items listed below.

Your article may not currently address all the items on the checklist. Please modify your text to include the missing information. If you are certain that an item does not apply, please write "n/a" and provide a short explanation.

Upload your completed checklist as an extra file when you submit to a journal.

In your methods section, say that you used the PRISMA-Preporting guidelines, and cite them as:

Moher D, Shamseer L, Clarke M, Ghersi D, Liberati A, Petticrew M, Shekelle P, Stewart LA. Preferred Reporting Items for Systematic Review and Meta-Analysis Protocols (PRISMA-P) 2015 statement. Syst Rev. 2015;4(1):1.

		Reporting Item	Page Number
**Title**			
Identification	#1a	Identify the report as a protocol of a systematic review	1
Update	#1b	If the protocol is for an update of a previous systematic review, identify as such	n/a

For peer review only

**Registration**			
	#2	If registered, provide the name of the registry (such as PROSPERO) and registration number	3
**Authors**			
Contact	#3a	Provide name, institutional affiliation, e-mail address of all protocol authors; provide physical mailing address of corresponding author	1
Contribution	#3b	Describe contributions of protocol authors and identify the guarantor of the review	18
**Amendments**			
	#4	If the protocol represents an amendment of a previously completed or published protocol, identify as such and list changes; otherwise, state plan for documenting important protocol amendments	n/a
**Support**			
Sources	#5a	Indicate sources of financial or other support for the review	18
Sponsor	#5b	Provide name for the review funder and / or sponsor	18
Role of sponsor or funder	#5c	Describe roles of funder(s), sponsor(s), and / or institution(s), if any, in developing the protocol	18
**Introduction**			

For peer review only

Rationale	#6	Describe the rationale for the review in the context of what is already known	4,5
Objectives	#7	Provide an explicit statement of the question(s) the review will address with reference to participants, interventions, comparators, and outcomes (PICO)	6
**Methods**			
Eligibility criteria	#8	Specify the study characteristics (such as PICO, study design, setting, time frame) and report characteristics (such as years considered, language, publication status) to be used as criteria for eligibility for the review	7-10
Information sources	#9	Describe all intended information sources (such as electronic databases, contact with study authors, trial registers or other grey literature sources) with planned dates of coverage	10
Search strategy	#10	Present draft of search strategy to be used for at least one electronic database, including planned limits, such that it could be repeated	10-11
Study records - data management	#11a	Describe the mechanism(s) that will be used to manage records and data throughout the review	11
Study records - selection process	#11b	State the process that will be used for selecting studies (such as two independent reviewers) through each phase of the review (that is, screening, eligibility and inclusion in meta-analysis)	11-12

For peer review only

Study records - data collection process	#11c	Describe planned method of extracting data from reports (such as piloting forms, done independently, in duplicate), any processes for obtaining and confirming data from investigators	12-13
Data items	#12	List and define all variables for which data will be sought (such as PICO items, funding sources), any pre-planned data assumptions and simplifications	12-13
Outcomes and prioritization	#13	List and define all outcomes for which data will be sought, including prioritization of main and additional outcomes, with rationale	13-14
Risk of bias in individual studies	#14	Describe anticipated methods for assessing risk of bias of individual studies, including whether this will be done at the outcome or study level, or both; state how this information will be used in data synthesis	14
Data synthesis	#15a	Describe criteria under which study data will be quantitatively synthesised	14-15
Data synthesis	#15b	If data are appropriate for quantitative synthesis, describe planned summary measures, methods of handling data and methods of combining data from studies, including any planned exploration of consistency (such as I2, Kendall's τ)	14-15
Data synthesis	#15c	Describe any proposed additional analyses (such as sensitivity or subgroup analyses, meta-regression)	n/a

For peer review only

Data synthesis	#15d	If quantitative synthesis is not appropriate, describe the type of summary planned	15
Meta-bias(es)	#16	Specify any planned assessment of meta-bias(es) (such as publication bias across studies, selective reporting within studies)	n/a
Confidence in cumulative evidence	#17	Describe how the strength of the body of evidence will be assessed (such as GRADE)	n/a

None The PRISMA-P checklist is distributed under the terms of the Creative Commons Attribution License CC-BY 4.0. This checklist can be completed online using https://www.goodreports.org/, a tool made by the EQUATOR Network in collaboration with Penelope.ai

For peer review only

The identification and prevalence of frailty in diabetes mellitus and association with clinical outcomes: A systematic review protocol

Journal:	*BMJ Open*
Manuscript ID	bmjopen-2020-037476.R1
Article Type:	Protocol
Date Submitted by the Author:	28-Apr-2020
Complete List of Authors:	Hanlon, Peter; University of Glasgow Institute of Health and Wellbeing, ;<br><br>Fauré, Isabella ; University of Glasgow Institute of Health and Wellbeing<br>Corcoran, Neave ; University of Glasgow Institute of Health and Wellbeing<br>Butterly, Elaine; University of Glasgow Institute of Health and Wellbeing<br>Lewsey, Jim; University of Glasgow Institute of Health and Wellbeing<br>McAllister, David; University of Glasgow Institute of Health and Wellbeing<br>Mair, Frances; University of Glasgow Institute of Health and Wellbeing
**Primary Subject Heading**:	Diabetes and endocrinology
Secondary Subject Heading:	General practice / Family practice, Geriatric medicine
Keywords:	Diabetes & endocrinology < INTERNAL MEDICINE, EPIDEMIOLOGY, GERIATRIC MEDICINE

I, the Submitting Author has the right to grant and does grant on behalf of all authors of the Work (as defined in the below author licence), an exclusive licence and/or a non-exclusive licence for contributions from authors who are: i) UK Crown employees; ii) where BMJ has agreed a CC-BY licence shall apply, and/or iii) in accordance with the terms applicable for US Federal Government officers or employees acting as part of their official duties; on a worldwide, perpetual, irrevocable, royalty-free basis to BMJ Publishing Group Ltd ("BMJ") its licensees and where the relevant Journal is co-owned by BMJ to the co-owners of the Journal, to publish the Work in this journal and any other BMJ products and to exploit all rights, as set out in our licence.

The Submitting Author accepts and understands that any supply made under these terms is made by BMJ to the Submitting Author unless you are acting as an employee on behalf of your employer or a postgraduate student of an affiliated institution which is paying any applicable article publishing charge ("APC") for Open Access articles. Where the Submitting Author wishes to make the Work available on an Open Access basis (and intends to pay the relevant APC), the terms of reuse of such Open Access shall be governed by a Creative Commons licence – details of these licences and which Creative Commons licence will apply to this Work are set out in our licence referred to above.

Other than as permitted in any relevant BMJ Author's Self Archiving Policies, I confirm this Work has not been accepted for publication elsewhere, is not being considered for publication elsewhere and does not duplicate material already published. I confirm all authors consent to publication of this Work and authorise the granting of this licence.

For peer review only

The identification and prevalence of frailty in diabetes mellitus and association with clinical outcomes: A systematic review protocol

For peer review only

Authors:

Dr Peter Hanlon[1]
Ms Isabella Fauré[1]
Dr Neave Corcoran[1]
Dr Elaine Butterly[2]
Professor Jim Lewsey[3]
Dr David McAllister[2]*
Professor Frances S Mair[1]*

*Joint senior author

Affiliations:

1. General Practice and Primary Care, Institute of Health and Wellbeing, University of Glasgow, Glasgow, UK
2. Public Health, Institute of Health and Wellbeing, University of Glasgow, Glasgow, UK
3. Health Economics and Health Technology Assessment, Institute of Health and Wellbeing, University of Glasgow, Glasgow, UK

Corresponding author:

Dr Peter Hanlon
General Practice and Primary Care
Institute of Health and Wellbeing
University of Glasgow
1 Horselethill Road
Glasgow, G12 9LX

For peer review only - http://bmjopen.bmj.com/site/about/guidelines.xhtml

28 Peter.hanlon@glasgow.ac.uk
29 +44 141 330 8383

30 Word count: 2311

31 **Keywords:** Frailty, Diabetes Mellitus, Systematic review, Protocol

For peer review only

For peer review only - http://bmjopen.bmj.com/site/about/guidelines.xhtml

Abstract

Introduction:

Diabetes mellitus is common and growing in prevalence, and an increasing proportion of people with diabetes are living to older age. Frailty is therefore becoming an important concept in diabetes. Frailty is associated with older age and describes a state of increased susceptibility to decompensation in response to physiological stress. A range of measures have been used to quantify frailty. This systematic review aims to identify measures used to quantify frailty in people with diabetes (type 1, type 2, or unspecified); to summarize the prevalence of frailty in diabetes; and to describe the relationship between frailty and adverse clinical outcomes in people with diabetes.

Methods and analysis:

Three electronic databases (Medline, Embase and Web of Science) will be searched from 2000 to November 2019 and supplemented by citation searching of relevant articles and hand-searching of reference lists. Two reviewers will independently review titles, abstracts and full texts. Inclusion criteria include: (1) Adults with diabetes mellitus (type 1, type 2, or unspecified); (2) Quantify frailty using any validated frailty measure; (3) Report the prevalence of frailty and/or the association between frailty and clinical outcomes in people with diabetes; (4) Studies that assess generic (e.g. mortality, hospital admission, falls) or diabetes specific outcomes (e.g. hypoglycaemic episodes, cardiovascular events, diabetic nephropathy, diabetic retinopathy); (5) Cross-sectional and longitudinal observational studies. Study quality will be assessed using the Newcastle-Ottawa scale for observational studies. Clinical and methodological heterogeneity will be assessed, and a random effects meta-analysis performed if appropriate. Otherwise, a narrative synthesis will be performed.

For peer review only - http://bmjopen.bmj.com/site/about/guidelines.xhtml

Ethics and dissemination:

This study will summarise current knowledge about measurement, prevalence and clinical implications of frailty in diabetes. This will inform future research and clinical guidelines to assess the balance of risks and treatment priorities in the growing number of people living with frailty and diabetes.

Registration number:

PROSPERO CRD42020163109.

Strengths and Limitations

This systematic review will provide a comprehensive overview of the prevalence and implications of frailty in people with diabetes.

We will include a broad range of frailty definitions and clinical outcomes relevant to diabetes.

There is likely to be significant heterogeneity between population characteristics and frailty definitions in included studies.

By including only English language articles, there is a chance of language bias in the results of the review.

We exclude Grey literature, which may lead to publication bias.

For peer review only

For peer review only - http://bmjopen.bmj.com/site/about/guidelines.xhtml

Introduction

Diabetes mellitus (hereafter "diabetes") describes a collection of metabolic disorders, with distinct pathological processes, that are characterised by elevated blood glucose.(1) The most common are type 1 diabetes and type 2 diabetes. Type 1 diabetes is caused by insulin deficiency resulting from destruction of pancreatic beta cells, usually by an autoimmune process.(2) Type 2 diabetes describes a relative insulin deficiency caused by beta-cell dysfunction and insulin resistance of target organs.(2) Both are associated with a range of complications including macrovascular disease, retinopathy, nephropathy and neuropathy.(3) The prevalence of diabetes is increasing across the world.(4) Population demographics are also shifting towards an ageing population.(5) Among people above the age of 65, the prevalence of diabetes can be as high as 30%.(6) Diabetes in older people is therefore a growing clinical and public health priority. One factor with important implications for disease management in older age is frailty.(7)

Frailty is a state characterised by reduced functional reserve across multiple physiological systems.(8) People living with frailty have impaired resolution of homeostasis following physiological stressors.(8) Frailty therefore carries an increased risk of a range of adverse health outcomes, such as falls, cognitive decline, hospital admission and mortality.(9) Frailty is widely recognised to be a multidimentional and dynamic state, associated with older age and with a range of non-communicable diseases.(9) However, there is no single universally accepted operational definition of frailty. Rather, a wide range of definitions have been utilized in both research and clinical practice.(10)

The two dominant paradigms in the frailty literature are the frailty phenotype and the frailty index. The frailty phenotype, described by *Fried et al* in 2001, defines frailty as the presence of three or more out of five features: low hand-grip strength, unintentional weight loss, low physical activity, exhaustion, and slow walking pace.(11) The presence of one or two of these features is classified as

For peer review only - http://bmjopen.bmj.com/site/about/guidelines.xhtml

a pre-frail state. The frailty index, described by Rockwood and Mitnitski in 2007, is based on a cumulative-deficit model of frailty whereby frailty is identified by counting the number of health ‘deficits’ present in an individual.(12) At least 30 deficits are required to construct a frailty index, all of which must increase in prevalence with age, be associated with poor health, and not saturate too early (i.e. be universally present among older people).(13) Both the frailty phenotype and frailty index have been associated with adverse health outcomes in a range of older populations, however the populations identified as frail by each are different.(14) Since their original description, a wide range of other frailty instruments, as well as adaptations of the frailty index and phenotype, have been developed for both epidemiological studies and for clinical practice.(9, 10)

The relationship between diabetes and frailty is complex. Diabetes is associated with a higher prevalence of frailty.(15-18) Both type 1 and type 2 diabetes lead to microvascular and macrovascular complications which have important physical, cognitive and functional consequences, that may contribute to the development of frailty.(6) Hyperglycaemia is also recognized to directly impact muscle mass and quality, exacerbating age-related sarcopenia and, in turn, physical function.(19) However, the association between frailty and poor functional outcomes in people with diabetes is only partially explained by direct complications of diabetes.(17, 20)

The importance of frailty in the context of diabetes is increasingly recognised in clinical guidelines.(7) Specifically, higher HbA1c targets are recommended in the context of frailty, in part due to the increased risks associated with hypoglycaemia.(21) Despite this, up to 40% of older people with diabetes may be over-treated (with HbA1c <7%).(22, 23) Conversely, poor glycaemic control and associated vascular complications risk causing, or accelerating the progression of, frailty.(24)

One recent meta-analysis demonstrated a consistent relationship between frailty and mortality, hospitalisation, and cardiovascular events in the context of diabetes.(25) We are not aware of any systematic review to assess the prevalence of frailty in diabetes, or to consider a broader range of outcomes relevant to the management of diabetes. Given the risks of both over- and under-

For peer review only - http://bmjopen.bmj.com/site/about/guidelines.xhtml

treatment of diabetes in the context of frailty, understanding the range of potential associations is required to inform clinical decisions and to underpin future research.

To enhance understanding of the implications and management of diabetes within an ageing population, it is important to fully describe the association between diabetes and frailty. Given the risks of both over- and under-treatment of diabetes in the context of frailty, it is important to understand the associations between frailty and a range of potential outcomes in diabetes. This includes generic outcomes such as mortality and hospitalisation and disability and disease specific outcomes such as retinopathy, neuropathy, and hypoglycaemic events. An understanding of the range and complexity of these associations is required to inform clinical decisions around treatment priorities and to underpin future research. This includes quantifying the prevalence of frailty in people with diabetes, and the impact that different frailty definitions might have on this prevalence. This manuscript describes the protocol of a systematic review aiming to synthesise existing evidence relating to these questions.

Aims

The systematic review will aim to:

- Identify which frailty measures have been used to assess frailty in people with diabetes (type1, type 2, or mixed/unspecified)
- Quantify the prevalence of frailty among people with diabetes
- Describe the association between frailty and both generic (e.g. mortality) and disease specific (e.g. hypoglycaemia) clinical outcomes in the context of diabetes

For peer review only - http://bmjopen.bmj.com/site/about/guidelines.xhtml

Methods and analysis

This protocol is registered with PROSPERO (CRD42020163109). The review will be conducted and reported according to the Preferred Reporting Items for Systematic Reviews and Meta-Analyses (PRISMA) statement.(26) Where a meta-analysis is undertaken, we will report findings according to the Meta-analyses Of Observational Studies in Epidemiology checklist.

Eligibility criteria for inclusion

The eligibility criteria for this review are summarized in table 1 and explained in more detail below.

Table 1. Inclusion Criteria

PECOS component	Description
Population	Adults (≥ 18 years old)<br>Diabetes (type 1, type 2, or unspecified)
Exposure	Frailty as assessed by any frailty measure
Comparator	People with diabetes not classified as frail
Outcomes	Generic:<br>• Mortality<br>• Major Adverse Cardiovascular Events<br>• Hospital admission<br>• Admission to long-term care facility<br>• Falls<br>• Number of clinic attendances<br>• Quality of life<br>• Disability/functional status<br>Diabetes specific:

For peer review only

For peer review only - http://bmjopen.bmj.com/site/about/guidelines.xhtml

	• HbA1c (cross sectional association, or longitudinal)<br>• Glycaemic variability<br>• Hypoglycaemic episodes<br>• Diabetic retinopathy (cross sectional association, or longitudinal)<br>• Diabetic nephropathy (cross sectional association, or longitudinal)<br>○ Include development of end-stage renal disease<br>• Diabetic foot complications (cross sectional association, or longitudinal)<br>• Treatment burden (e.g. Diabetic Treatment Burden Questionnaire)
Settings	Community (including care home/nursing home)<br>Outpatient clinic<br>Inpatient
Study design	Cross sectional or longitudinal<br>Cohort
Other exclusions	Conference abstracts, letters, review articles, intervention studies, Grey literature

For peer review only

Population

We will include studies analysing data from people with any form of diabetes.

While frailty is a state associated with increasing age, there is evidence that frailty is identifiable in relatively younger people, particularly in certain contexts such as multimorbidity (2 or more co-existing long-term conditions) or in areas of high socioeconomic deprivation. We will therefore include studies of adults of any age (≥18 years). However, we anticipate that most studies will focus predominantly on 'older' populations.

For peer review only - http://bmjopen.bmj.com/site/about/guidelines.xhtml

153 From an initial scoping of the literature, it is likely that many studies describing frailty in population-based studies measure unspecified 'diabetes' rather than explicitly type 1 or type 2 diabetes. We will therefore include any study which includes people with type 1, type 2 diabetes, or people with unspecified diabetes. Given that frailty is a state associated with older age, and that type 2 diabetes is both more prevalent than type 1 diabetes and becomes more prevalent with age, it is likely that most (but not all) people with diabetes in the relevant populations will have type 2 diabetes. Studies of type 1 diabetes, type 2 diabetes and those of unspecified diabetes will be considered separately in any subsequent analysis.

We will include studies focusing purely on people with diabetes, or population-based studies that report results for people with diabetes separately.

Exposure

The 'exposure' of interest is frailty. Many epidemiological measures and clinical tools have been developed to identify frailty for research or clinical practice.(10)

To be eligible for inclusion, a study must use a measure which explicitly seeks to quantify frailty. We will include measures developed primarily as epidemiological tools (e.g. the frailty phenotype frailty index).(11, 12) We will also include measures designed primarily for clinical practice (e.g. the Clinical Frailty Scale).(27)

Studies focusing solely on comorbidity (i.e. no additional measures to identify 'frailty') will be excluded unless these are explicitly operationalised as a 'frailty index'. In this case studies would generally be expected to include additional deficits (such as symptoms, functional limitations, laboratory measures etc.). Studies which use a single parameter as a proxy for frailty (e.g. grip strength alone, self-rated health) will be excluded.

For peer review only - http://bmjopen.bmj.com/site/about/guidelines.xhtml

Comparator

Studies that report the prevalence of frailty will be eligible for inclusion if they report the prevalence of frailty in diabetes only. Studies should report the number or proportion of participants with and without frailty (or with varying degrees of frailty, depending on the measure used).

For assessing the association between frailty and clinical outcomes in the context of diabetes, studies should report the association between frailty and the outcome of interest. This may be reported either as the association with the presence or absence of frailty (in the case of a binary or categorical measure) or the association between the degree of frailty and the outcome (in the case of a continuous or ordinal measure of frailty).

Outcomes

Outcomes of interest are summarized in table 1. We will include studies assessing any of these outcomes as long as the association is specifically quantified in people with diabetes and frailty.

Setting

We will include studies of community-dwelling patients, outpatient populations or hospital inpatients.

For the purposes of this review, given the focus on frailty, people living in long-term care facilities (e.g. care-homes, nursing-homes) will be considered to be ‘community-dwelling’. Therefore, any study including, or specifically recruiting, nursing home residents will be eligible for inclusion.

For peer review only

For peer review only - http://bmjopen.bmj.com/site/about/guidelines.xhtml

Identification of studies

Electronic searches

Medline, Embase, and Web of Science (Core collection) databases will be search using a combination of Medical Subject Headings (MeSH) and keyword searches (Supplementary file 1). The terms used for the medline search are shown in table 2. These terms will be adapted for the other databases. Searches will be from 2000 to November 2019. The year 2000 was chosen as the start date as the first seminal paper operationalising the concept of frailty in an epidemiological study was published in 2001. Articles published prior to this date are therefore unlikely to be relevant. No language restriction will be applied to the search, but only English language articles will be included at the screening level. This language restriction is a pragmatic decision, however we acknowledge that this may lead to a language bias in the results, potentially excluding relevant studies published in other languages.

Table 2: Medline Search
1. Exp Frailty/<br>2. Exp Frail Elderly/<br>3. Frail*.tw<br>4. 1 or 2 or 3<br>5. Exp Diabetes Mellitus<br>6. Diabet*.tw<br>7. (IDDM or NIDDM or MODY or T1DM, or T2DM or T1D or T2D).tw<br>8. (non insulin* depend* or non insulin depend* or non insulin?depend* or non insulin ?depend).tw<br>9. (insulin* depend* or insulin ?depend*).tw<br>10. 5 or 6 or 7 or 8 or 9

For peer review only - http://bmjopen.bmj.com/site/about/guidelines.xhtml

For peer review only

11. Exp Diabetes Insipidus/
12. Diabet* insipidus.tw
13. 11 or 12
14. 10 not 13
15. 4 and 14

205 ## Identifying additional articles

206 Electronic searches will be supplemented by hand searching reference lists of relevant articles. A
207 citation search of all relevant articles will also be carried out using the Web of Science citation search
208 tool.

209 ## Data collection and analysis

210 ### Selection of studies

211 Two reviewers, working independently, will screen all titles and abstracts of records identified in the
212 database searches. PECOS criteria outlined above will be used to determine eligibility. Where there
213 is disagreement, studies will be retained for full-text screening.

214 Full texts of all potentially eligible studies will be screened independently by two reviewers.
215 Disagreements about eligibility will be resolved by consensus, involving a third reviewer where
216 necessary.

217 ### Data extraction

218 A standard data extraction form will be designed and piloted before being applied to each of the
219 included studies. Extracted data will include:

220 Study details

For peer review only - http://bmjopen.bmj.com/site/about/guidelines.xhtml

- Author
- Year
- Location
- Setting (community, outpatient, residential care)
- Method of recruitment (e.g. random sample, postal invitation, consecutive patients)
- Method of assessment (face-to-face, survey, linkage to healthcare records)

Population

- Age
- Sex
- Ethnicity
- Socioeconomic status
- Comorbidities
- Medications
- Social circumstances (e.g. living independently, requiring carers, family support etc)
- Smoking status
- Physical activity

Diabetes details

- Type of diabetes (type 1, type 2 or unspecified)
- Method of confirmation (self-report, medical records, clinical assessment)
- Measure of control (e.g. HbA1c)
- Medication (e.g. proportion taking insulin, oral antidiabetics etc.)
- Presence and severity of complications (e.g. retinopathy, nephropathy, neuropathy, ulceration, Charcot arthropathy)

Frailty definition

For peer review only

For peer review only - http://bmjopen.bmj.com/site/about/guidelines.xhtml

- Frailty measure used
- Definitions for each component of the frailty measure (e.g. cut-points used for continuous measures, method of assessment (questionnaire, interview etc.))

Frailty prevalence

Outcomes (generic):

- Mortality
- Major Adverse Cardiovascular Events
- Hospital admission
- Admission to long-term care facility
- Falls
- Number of clinic attendances
- Quality of life
- Disability/functional status

Outcomes (diabetes specific):

- HbA1c (cross sectional association, or longitudinal)
- Glycaemic variability
- Hypoglycaemic episodes
- Diabetic retinopathy (cross sectional association, or longitudinal)
- Diabetic nephropathy (cross sectional association, or longitudinal)
- Diabetic foot complications (cross sectional association, or longitudinal)
- Treatment burden (e.g. Diabetic Treatment Burden Questionnaire)

As we include a wide range of outcomes, it is likely that the way outcomes are assessed will vary depending on the outcome in question. Studies may also assess similar outcomes (e.g. hospital admission) in different ways (e.g. number of admissions over specified follow-up, time to first

For peer review only

For peer review only - http://bmjopen.bmj.com/site/about/guidelines.xhtml

admission, presence of absence of admission during follow-up). For the outcomes listed above, we will extract data regardless of the method of assessment. Heterogeneity in the way outcome data were collected will be used to inform the approach to data synthesis (i.e. meta-analysis versus narrative synthesis). For each outcome reported we will record

- The method of outcome assessment (e.g. linkage to healthcare records, face-to-face assessment, questionnaire etc.)
- Method of analysis (e.g. time-to-event, mean difference etc.)
- The association between frailty and the outcome (e.g. prevalence, odds ratio, hazard ratio etc.)
- Adjustment for any potential confounders
- Length of follow-up over which the outcome was assessed
- Method of analysis of competing risks when assessing each outcome.

Where available, we will also extract data on both relative (e.g. hazard ratios) and absolute (e.g. events per 1000 people) associations with outcomes.

Assessment of methodological quality

The Newcastle-Ottawa scale will be used to assess the risk of bias for each study (Supplementary file 2).(28) This scale is widely used for the assessment of observational studies, and has frequently been adapted to the context of specific systematic reviews. We have adapted the criteria in order to be explicit about how the ‘exposure assessment’ related to frailty: specifically, awarding one point for the use of a validated frailty assessment measure. For cross-sectional studies, only the first 5 elements of the scale were relevant to quality assessment (the remainder concerning the longitudinal assessment of outcomes). We will use this subsection of the Newcastle-Ottawa scale to assess the quality of cross-sectional studies to allow direct comparability with the baseline assessments of longitudinal studies (from which we will also extract data on frailty prevalence). In

For peer review only - http://bmjopen.bmj.com/site/about/guidelines.xhtml

293 assessing the comparability of frail/non-frail groups, age will be taken as the most important factor
294 for which studies should account.

295 Data synthesis

296 The appropriate method of data synthesis will be determined after assessment of the heterogeneity
297 of the included studies, in terms of population selection and demographics, frailty definition, and
298 method of outcome assessment.

299 With regards to the prevalence of frailty, different frailty measures will be considered separately (i.e.
300 we will not perform a meta-analysis of frailty prevalence measured using different scales). We will
301 also consider community studies separately from studies focussing on outpatient clinic populations
302 (as these may represent people with more severe diabetes), inpatients or people living in residential
303 care. We will also assess the inclusion criteria and demographics of the sample population, with
304 particular attention to age (as frailty is strongly associated with age) and sex (as women tend to have
305 a higher prevalence of frailty than men) to determine the most appropriate method of synthesis.
306 Where samples have been drawn from populations with a markedly different age/sex structure, a
307 pooled estimate of the mean prevalence of frailty across these studies is unlikely to be a meaningful
308 summary. Similarly, other inclusion criteria used by the individual studies (such as excluding
309 'institutionalised' people, people with cognitive impairment, of people with impaired mobility
310 unable to attend an assessment) may disproportionately impact on the estimation of frailty
311 prevalence. The appropriateness, or otherwise, of a meta-analysis of frailty prevalence will be
312 judged only after examination of these aspects of the included studies.

313 For the assessment of outcomes, the approach to synthesis will also be judged based on
314 heterogeneity of the method of outcome assessment and the analytic approach. As above, different
315 frailty measures will be considered separately.

For peer review only - http://bmjopen.bmj.com/site/about/guidelines.xhtml

If appropriate, we will combine these in a random effects meta-analysis (anticipating heterogeneity in the true association). As well as a pooled estimate and 95% confidence intervals, we will also calculate the prediction interval to assess the range of plausible estimates from the observed data. Heterogeneity will be quantified using the I^2 statistic. Where heterogeneity is present, we will attempt to explore potential sources of heterogeneity using subgroup analyses (e.g. by method of determining frailty, age of sample population, method of outcome assessment). By doing so, we propose to explore factors that may influence the estimates reported in observational studies in the presence of heterogeneity, rather than provide a definitive single estimate.(29) We will use funnel plots to assess for potential publication bias.

Only those studies that are judged to be sufficiently comparable will be included in meta-analyses. For outcomes where there are too few studies, or the included studies are too heterogenous to permit a meaningful meta-analysis (for example, in terms of outcome definition or method of assessing frailty), we will perform a narrative synthesis of the study findings. This will report the methods used to identify frailty along with the prevalence and association with outcomes, to explore the impact of the method of assessment on the observed relationship. This will be reported alongside detail of the recruitment strategy, age profile, and characteristics of each sample included.

Patient and public involvement

No patients were involved in the development of this review.

For peer review only

For peer review only - http://bmjopen.bmj.com/site/about/guidelines.xhtml

Ethics and dissemination

This systematic review will provide an overview of the prevalence of frailty in diabetes, and the relationship between frailty and adverse health outcomes in people with diabetes.

As the prevalence of both frailty and diabetes increase, it will become increasingly important for clinical guidelines for the treatment of diabetes to explicitly consider the needs of people living with frailty.(7) Quantifying the prevalence of frailty in diabetes will allow the scale of this challenge to be better appreciated. By including any reported definition of frailty within our inclusion criteria, this review will demonstrate which of the wide range of frailty instruments and measures have been used to study frailty in diabetes. It will also be possible to compare if and how prevalence and association with outcomes differs depending on the frailty definition used.

Given the likely heterogeneity in frailty definitions, as well as inherent differences in the populations studied, it may not be possible to undertake a meta-analysis of the findings of this review. If this is the case, we propose to conduct a detailed narrative synthesis, systematically describing and synthesizing details of the populations under study as well as the details of frailty definitions used.

We also propose to search for and extract data for a wide range of clinical outcomes. Given the multidimentional nature of frailty,(8) and the vulnerability to decompensation that is inherent to any frailty definition,(9) it is likely that frailty will be associated with a range of adverse outcomes. The challenge in translating these associations into meaningful recommendations is understanding the balance of these risks, and how they might inform clinical decisions and recommendations. The balance of risks in diabetes, and treatment priorities, may differ depending on the degree of frailty experienced by an individual. The associations may also differ in their nature or magnitude depending on the method used to identify frailty. This review will aim to provide an overview of what is known about the relationship between frailty and both generic and disease specific

For peer review only - http://bmjopen.bmj.com/site/about/guidelines.xhtml

outcomes. This is likely to inform priorities for future research into the consequences of frailty in diabetes.

As this project is a systematic review, ethical approval is not required. Patients or the public were not involved in the development of this protocol.

For peer review only

For peer review only - http://bmjopen.bmj.com/site/about/guidelines.xhtml

Author contributions

All authors (PH, IF, NC, EB, JL, DM and FM) contributed to the conception and design of the proposed study. PH, DM and FM developed the data sources and search strategy. PH, IF, NC, DM and FM refined the inclusion criteria. PH, IF, NC, DM and FM developed the data extraction template which was piloted by PH, IF and NC. PH and IF wrote the first draft. All authors critically reviewed this and subsequent drafts. All authors approved the final version of the manuscript for submission. FM is the guarantor of the review. All authors accept accountability for the accuracy of the protocol.

Funding statement

Dr Peter Hanlon was funded by a Medical Research Council Clinical Research Training Fellowship (Grant reference MR/S021949/1) entitled "Understanding prevalence and impact of frailty in chronic disease and implications for clinical management. The funder had no role in protocol development.

Competing interests statement

The authors declare no competing interests.

For peer review only

References

1. Moini J. Epidemiology of Diabetes: Elsevier; 2019.

2. Ozougwu J, Obimba K, Belonwu C, Unakalamba C. The pathogenesis and pathophysiology of type 1 and type 2 diabetes mellitus. Journal of physiology and pathophysiology. 2013;4(4):46-57.

3. Pambianco G, Costacou T, Ellis D, Becker DJ, Klein R, Orchard TJ. The 30-year natural history of type 1 diabetes complications: the Pittsburgh Epidemiology of Diabetes Complications Study experience. Diabetes. 2006;55(5):1463-9.

4. Shaw JE, Sicree RA, Zimmet PZ. Global estimates of the prevalence of diabetes for 2010 and 2030. Diabetes research and clinical practice. 2010;87(1):4-14.

5. United Nations. World Population Ageing [Report] 2015 [Available from: https://www.un.org/en/development/desa/population/publications/pdf/ageing/WPA2015_Report.pdf.

6. Sinclair AJ, Rodriguez-Manas L. Diabetes and Frailty: Two Converging Conditions? Canadian journal of diabetes. 2016;40(1):77-83.

7. Sinclair AJ, Abdelhafiz A, Dunning T, Izquierdo M, Manas LR, Bourdel-Marchasson I, et al. An international position statement on the management of frailty in diabetes mellitus: summary of recommendations 2017. The Journal of frailty & aging. 2018;7(1):10-20.

8. Clegg A, Young J, Iliffe S, Rikkert MO, Rockwood K. Frailty in elderly people. The Lancet. 2013;381(9868):752-62.

9. Hoogendijk EO, Afilalo J, Ensrud KE, Kowal P, Onder G, Fried LP. Frailty: implications for clinical practice and public health. The Lancet. 2019;394(10206):1365-75.

10. Bouillon K, Kivimaki M, Hamer M, Sabia S, Fransson EI, Singh-Manoux A, et al. Measures of frailty in population-based studies: an overview. BMC Geriatrics. 2013;13(1):64.

11. Fried LP, Tangen CM, Walston J, Newman AB, Hirsch C, Gottdiener J, et al. Frailty in Older Adults: Evidence for a Phenotype. The Journals of Gerontology: Series A. 2001;56(3):M146-M57.

12. Rockwood K, Mitnitski A. Frailty in Relation to the Accumulation of Deficits. The Journals of Gerontology: Series A. 2007;62(7):722-7.

13. Searle SD, Mitnitski A, Gahbauer EA, Gill TM, Rockwood K. A standard procedure for creating a frailty index. BMC Geriatrics. 2008;8(1):24.

14. Santos-Eggimann B, Sirven N. Screening for frailty: older populations and older individuals. Public health reviews. 2016;37(1):7.

15. Kotsani M, Chatziadamidou T, Economides D, Benetos A. Higher prevalence and earlier appearance of geriatric phenotypes in old adults with type 2 diabetes mellitus. Diabetes Research and Clinical Practice. 2018;135:206-17.

16. Thein FS, Li Y, Nyunt MSZ, Gao Q, Wee SL, Ng TP. Physical frailty and cognitive impairment is associated with diabetes and adversely impact functional status and mortality. Postgraduate medicine. 2018;130(6):561-7.

17. Castro-Rodriguez M, Carnicero JA, Garcia-Garcia FJ, Walter S, Morley JE, Rodriguez-Artalejo F, et al. Frailty as a Major Factor in the Increased Risk of Death and Disability in Older People With Diabetes. Journal of the American Medical Directors Association. 2016;17(10):949-55.

18. Hanlon P, Hannigan L, Fischbacker C, Welton N, Dias S, Mair F, et al. Representation of people with comorbidity and multimorbidity in clinical trials of novel drug therapies; an individual-level participant data analysis. BMC Medicine (in press). 2019.

19. Yoon JW, Ha Y-C, Kim KM, Moon JH, Choi SH, Lim S, et al. Hyperglycemia Is Associated with Impaired Muscle Quality in Older Men with Diabetes: The Korean Longitudinal Study on Health and Aging. Diabetes Metab J. 2016;40(2):140-6.

20. Maggi S, Noale M, Gallina P, Marzari C, Bianchi D, Limongi F, et al. Physical disability among older Italians with diabetes. The ILSA Study. Diabetologia. 2004;47(11):1957-62.

For peer review only

For peer review only - http://bmjopen.bmj.com/site/about/guidelines.xhtml

21. Schernthaner G, Schernthaner-Reiter MH. Diabetes in the older patient: heterogeneity requires individualisation of therapeutic strategies. Diabetologia. 2018;61(7):1503-16.

22. Formiga F, Franch-Nadal J, Rodriguez L, Ávila L, Fuster E. Inadequate glycaemic control and therapeutic management of adults over 65 years old with type 2 diabetes mellitus in Spain. The journal of nutrition, health & aging. 2017;21(10):1365-70.

23. Braun AK, Kubiak T, Kuntsche J, Meier-Höfig M, Müller UA, Feucht I, et al. SGS: a structured treatment and teaching programme for older patients with diabetes mellitus—a prospective randomised controlled multi-centre trial. Age and ageing. 2009;38(4):390-6.

24. Quartuccio M, Buta B, Kalyani RR. Comparative effectiveness for glycemic control in older adults with diabetes. Current geriatrics reports. 2017;6(3):175-86.

25. Ida S, Kaneko R, Imataka K, Murata K. Relationship between frailty and mortality, hospitalization, and cardiovascular diseases in diabetes: a systematic review and meta-analysis. Cardiovascular Diabetology. 2019;18(1):81.

26. Moher D, Liberati A, Tetzlaff J, Altman DG. Preferred reporting items for systematic reviews and meta-analyses: the PRISMA statement. BMJ. 2009;339:b2535.

27. Rockwood K, Song X, MacKnight C, Bergman H, Hogan DB, McDowell I, et al. A global clinical measure of fitness and frailty in elderly people. Canadian Medical Association Journal. 2005;173(5):489.

28. Stang A. Critical evaluation of the Newcastle-Ottawa scale for the assessment of the quality of nonrandomized studies in meta-analyses. European Journal of Epidemiology. 2010;25(9):603-5.

29. Mueller M, D'Addario M, Egger M, Cevallos M, Dekkers O, Mugglin C, et al. Methods to systematically review and meta-analyse observational studies: a systematic scoping review of recommendations. BMC Medical Research Methodology. 2018;18(1):44.

For peer review only

Medline Search Strategy

Search Terms

1. Exp Frailty/
2. Exp Frail Elderly/
3. Frail*.tw
4. 1 or 2 or 3
5. Exp Diabetes Mellitus
6. Diabet*.tw
7. (IDDM or NIDDM or MODY or T1DM, or T2DM or T1D or T2D).tw
8. (non insulin* depend* or non insulin depend* or non insulin?depend* or non insulin ?depend).tw
9. (insulin* depend* or insulin ?depend*).tw
10. 5 or 6 or 7 or 8 or 9
11. Exp Diabetes Insipidus/
12. Diabet* insipidus.tw
13. 11 or 12
14. 10 not 13
15. 4 and 14

Language restriction

None applied to search (non-English language studies excluded at screening stage)

Years searched

2001-November 2019

For peer review only

For peer review only

The Newcastle-Ottawa Scale

Adaptation for studies assessing the prevalence and impact of frailty in diabetes

1 – Representativeness of the exposed (i.e. frail) cohort

a) Truly representative (one star)

b) Somewhat representative (one star)

c) Selected group

d) No description of the derivation of the cohort

2 – Selection of the non-exposed (i.e. non-frail) cohort

a) Drawn from the same community as the exposed cohort (one star)

b) Drawn from a different source

c) No description of the derivation of the non-exposed cohort

3 – Ascertainment of exposure

a) Validated measurement tool for frailty (two stars)

b) Non-validated measurement tool, but the tool is available or described (one star)

c) No description of measurement tool

4 – Non-respondents

a) Comparability between respondents and non-respondents' characteristics is established, and the response rate is satisfactory (one star)

b) The response rate is unsatisfactory, or the comparability between respondents and non-respondents is unsatisfactory

c) No description of the response rate of the characteristics of the responders and non-responders

5 – Demonstration that outcome of interest was not present at the start of the study

a) Yes (one star)

b) No

Comparability:

1 – Comparability of the cohorts on the basis of the design or analysis being controlled for confounders

a) The study controls for age and sex (one star)

b) The study controls for other factors (one star)

For peer review only

c) Cohorts are not comparable on the basis of the design or analysis controlled for confounders

Outcomes:

1 – Assessment of outcomes

a) Independent assessment (one star)

b) Record linkage (one star)

c) Self-report

d) No description

e) Other

2 – Follow-up long enough for outcomes to occur

a) Yes (one star)

b) No

3 – Adequacy of follow-up of cohorts

a) Complete follow-up: all subjects accounted for (one star)

b) Subjects lost to follow-up unlikely to introduce bias – number lost less than or equal to 20% or description of those lost suggested no different from those followed (one star)

c) Follow-up rate less than 80% and no description of those lost

d) No statement

For peer review only

For peer review only

Reporting checklist for protocol of a systematic review.

Based on the PRISMA-P guidelines.

Instructions to authors

Complete this checklist by entering the page numbers from your manuscript where readers will find each of the items listed below.

Your article may not currently address all the items on the checklist. Please modify your text to include the missing information. If you are certain that an item does not apply, please write "n/a" and provide a short explanation.

Upload your completed checklist as an extra file when you submit to a journal.

In your methods section, say that you used the PRISMA-Preporting guidelines, and cite them as:

Moher D, Shamseer L, Clarke M, Ghersi D, Liberati A, Petticrew M, Shekelle P, Stewart LA. Preferred Reporting Items for Systematic Review and Meta-Analysis Protocols (PRISMA-P) 2015 statement. Syst Rev. 2015;4(1):1.

		Reporting Item	Page Number
**Title**			
Identification	#1a	Identify the report as a protocol of a systematic review	1
Update	#1b	If the protocol is for an update of a previous systematic review, identify as such	n/a

For peer review only

**Registration**			
	#2	If registered, provide the name of the registry (such as PROSPERO) and registration number	3
**Authors**			
Contact	#3a	Provide name, institutional affiliation, e-mail address of all protocol authors; provide physical mailing address of corresponding author	1
Contribution	#3b	Describe contributions of protocol authors and identify the guarantor of the review	18
**Amendments**			
	#4	If the protocol represents an amendment of a previously completed or published protocol, identify as such and list changes; otherwise, state plan for documenting important protocol amendments	n/a
**Support**			
Sources	#5a	Indicate sources of financial or other support for the review	18
Sponsor	#5b	Provide name for the review funder and / or sponsor	18
Role of sponsor or funder	#5c	Describe roles of funder(s), sponsor(s), and / or institution(s), if any, in developing the protocol	18
**Introduction**			

For peer review only

Rationale	#6	Describe the rationale for the review in the context of what is already known	4,5
Objectives	#7	Provide an explicit statement of the question(s) the review will address with reference to participants, interventions, comparators, and outcomes (PICO)	6
**Methods**			
Eligibility criteria	#8	Specify the study characteristics (such as PICO, study design, setting, time frame) and report characteristics (such as years considered, language, publication status) to be used as criteria for eligibility for the review	7-10
Information sources	#9	Describe all intended information sources (such as electronic databases, contact with study authors, trial registers or other grey literature sources) with planned dates of coverage	10
Search strategy	#10	Present draft of search strategy to be used for at least one electronic database, including planned limits, such that it could be repeated	10-11
Study records - data management	#11a	Describe the mechanism(s) that will be used to manage records and data throughout the review	11
Study records - selection process	#11b	State the process that will be used for selecting studies (such as two independent reviewers) through each phase of the review (that is, screening, eligibility and inclusion in meta-analysis)	11-12

For peer review only

Study records - data collection process	#11c	Describe planned method of extracting data from reports (such as piloting forms, done independently, in duplicate), any processes for obtaining and confirming data from investigators	12-13
Data items	#12	List and define all variables for which data will be sought (such as PICO items, funding sources), any pre-planned data assumptions and simplifications	12-13
Outcomes and prioritization	#13	List and define all outcomes for which data will be sought, including prioritization of main and additional outcomes, with rationale	13-14
Risk of bias in individual studies	#14	Describe anticipated methods for assessing risk of bias of individual studies, including whether this will be done at the outcome or study level, or both; state how this information will be used in data synthesis	14
Data synthesis	#15a	Describe criteria under which study data will be quantitatively synthesised	14-15
Data synthesis	#15b	If data are appropriate for quantitative synthesis, describe planned summary measures, methods of handling data and methods of combining data from studies, including any planned exploration of consistency (such as I2, Kendall's τ)	14-15
Data synthesis	#15c	Describe any proposed additional analyses (such as sensitivity or subgroup analyses, meta-regression)	n/a

For peer review only

Data synthesis	#15d	If quantitative synthesis is not appropriate, describe the type of summary planned	15
Meta-bias(es)	#16	Specify any planned assessment of meta-bias(es) (such as publication bias across studies, selective reporting within studies)	n/a
Confidence in cumulative evidence	#17	Describe how the strength of the body of evidence will be assessed (such as GRADE)	n/a

None The PRISMA-P checklist is distributed under the terms of the Creative Commons Attribution License CC-BY 4.0. This checklist can be completed online using https://www.goodreports.org/, a tool made by the EQUATOR Network in collaboration with Penelope.ai

For peer review only

BMJ Open

The identification and prevalence of frailty in diabetes mellitus and association with clinical outcomes: A systematic review protocol

Journal:	*BMJ Open*
Manuscript ID	bmjopen-2020-037476.R2
Article Type:	Protocol
Date Submitted by the Author:	23-Jul-2020
Complete List of Authors:	Hanlon, Peter; University of Glasgow Institute of Health and Wellbeing, ;<br><br>Fauré, Isabella ; University of Glasgow Institute of Health and Wellbeing<br>Corcoran, Neave ; University of Glasgow Institute of Health and Wellbeing<br>Butterly, Elaine; University of Glasgow Institute of Health and Wellbeing<br>Lewsey, Jim; University of Glasgow Institute of Health and Wellbeing<br>McAllister, David; University of Glasgow Institute of Health and Wellbeing<br>Mair, Frances; University of Glasgow Institute of Health and Wellbeing
**Primary Subject Heading**:	Diabetes and endocrinology
Secondary Subject Heading:	General practice / Family practice, Geriatric medicine
Keywords:	Diabetes & endocrinology < INTERNAL MEDICINE, EPIDEMIOLOGY, GERIATRIC MEDICINE

SCHOLARONE™
Manuscripts

I, the Submitting Author has the right to grant and does grant on behalf of all authors of the Work (as defined in the below author licence), an exclusive licence and/or a non-exclusive licence for contributions from authors who are: i) UK Crown employees; ii) where BMJ has agreed a CC-BY licence shall apply, and/or iii) in accordance with the terms applicable for US Federal Government officers or employees acting as part of their official duties; on a worldwide, perpetual, irrevocable, royalty-free basis to BMJ Publishing Group Ltd ("BMJ") its licensees and where the relevant Journal is co-owned by BMJ to the co-owners of the Journal, to publish the Work in this journal and any other BMJ products and to exploit all rights, as set out in our licence.

The Submitting Author accepts and understands that any supply made under these terms is made by BMJ to the Submitting Author unless you are acting as an employee on behalf of your employer or a postgraduate student of an affiliated institution which is paying any applicable article publishing charge ("APC") for Open Access articles. Where the Submitting Author wishes to make the Work available on an Open Access basis (and intends to pay the relevant APC), the terms of reuse of such Open Access shall be governed by a Creative Commons licence – details of these licences and which Creative Commons licence will apply to this Work are set out in our licence referred to above.

Other than as permitted in any relevant BMJ Author's Self Archiving Policies, I confirm this Work has not been accepted for publication elsewhere, is not being considered for publication elsewhere and does not duplicate material already published. I confirm all authors consent to publication of this Work and authorise the granting of this licence.

For peer review only

The identification and prevalence of frailty in diabetes mellitus and association with clinical outcomes: A systematic review protocol

Authors:

Dr Peter Hanlon[1]
Ms Isabella Fauré[1]
Dr Neave Corcoran[1]
Dr Elaine Butterly[2]
Professor Jim Lewsey[3]
Dr David McAllister[2]*
Professor Frances S Mair[1]*

*Joint senior author

Affiliations:

1. General Practice and Primary Care, Institute of Health and Wellbeing, University of Glasgow, Glasgow, UK
2. Public Health, Institute of Health and Wellbeing, University of Glasgow, Glasgow, UK
3. Health Economics and Health Technology Assessment, Institute of Health and Wellbeing, University of Glasgow, Glasgow, UK

Corresponding author:

Dr Peter Hanlon
General Practice and Primary Care
Institute of Health and Wellbeing
University of Glasgow
1 Horselethill Road
Glasgow, G12 9LX

For peer review only - http://bmjopen.bmj.com/site/about/guidelines.xhtml

Peter.hanlon@glasgow.ac.uk
+44 141 330 8383

Word count: 2311

Keywords: Frailty, Diabetes Mellitus, Systematic review, Protocol

For peer review only

For peer review only - http://bmjopen.bmj.com/site/about/guidelines.xhtml

Abstract

Introduction:

Diabetes mellitus is common and growing in prevalence, and an increasing proportion of people with diabetes are living to older age. Frailty is therefore becoming an important concept in diabetes. Frailty is associated with older age and describes a state of increased susceptibility to decompensation in response to physiological stress. A range of measures have been used to quantify frailty. This systematic review aims to identify measures used to quantify frailty in people with diabetes (any type); to summarize the prevalence of frailty in diabetes; and to describe the relationship between frailty and adverse clinical outcomes in people with diabetes.

Methods and analysis:

Three electronic databases (Medline, Embase and Web of Science) will be searched from 2000 to November 2019 and supplemented by citation searching of relevant articles and hand-searching of reference lists. Two reviewers will independently review titles, abstracts and full texts. Inclusion criteria include: (1) Adults with any type of diabetes mellitus; (2) Quantify frailty using any validated frailty measure; (3) Report the prevalence of frailty and/or the association between frailty and clinical outcomes in people with diabetes; (4) Studies that assess generic (e.g. mortality, hospital admission, falls) or diabetes specific outcomes (e.g. hypoglycaemic episodes, cardiovascular events, diabetic nephropathy, diabetic retinopathy); (5) Cross-sectional and longitudinal observational studies. Study quality will be assessed using the Newcastle-Ottawa scale for observational studies. Clinical and methodological heterogeneity will be assessed, and a random effects meta-analysis performed if appropriate. Otherwise, a narrative synthesis will be performed.

For peer review only - http://bmjopen.bmj.com/site/about/guidelines.xhtml

Ethics and dissemination:

This manuscript describes the protocol for a systematic review of observational studies and does not require ethical approval.

Registration number:

PROSPERO CRD42020163109.

Strengths and Limitations

This systematic review will provide a comprehensive overview of the prevalence and implications of frailty in people with diabetes.

We will include a broad range of frailty definitions and clinical outcomes relevant to diabetes.

There is likely to be significant heterogeneity between population characteristics and frailty definitions in included studies.

By including only English language articles, there is a chance of language bias in the results of the review.

We exclude Grey literature, which may lead to publication bias.

For peer review only

For peer review only - http://bmjopen.bmj.com/site/about/guidelines.xhtml

Introduction

Diabetes mellitus (hereafter "diabetes") describes a collection of metabolic disorders, with distinct pathological processes, that are characterised by elevated blood glucose.(1) The most common are type 1 diabetes and type 2 diabetes. Type 1 diabetes is caused by insulin deficiency resulting from destruction of pancreatic beta cells, usually by an autoimmune process.(2) Type 2 diabetes describes a relative insulin deficiency caused by beta-cell dysfunction and insulin resistance of target organs.(2) Both are associated with a range of complications including macrovascular disease, retinopathy, nephropathy and neuropathy.(3) The prevalence of diabetes is increasing across the world.(4) Population demographics are also shifting towards an ageing population.(5) Among people above the age of 65, the prevalence of diabetes can be as high as 30%.(6) Diabetes in older people is therefore a growing clinical and public health priority. One factor with important implications for disease management in older age is frailty.(7)

Frailty is a state characterised by reduced functional reserve across multiple physiological systems.(8) People living with frailty have impaired resolution of homeostasis following physiological stressors.(8) Frailty therefore carries an increased risk of a range of adverse health outcomes, such as falls, cognitive decline, hospital admission and mortality.(9) Frailty is widely recognised to be a multidimentional and dynamic state, associated with older age and with a range of non-communicable diseases.(9) However, there is no single universally accepted operational definition of frailty. Rather, a wide range of definitions have been utilized in both research and clinical practice.(10)

The two dominant paradigms in the frailty literature are the frailty phenotype and the frailty index. The frailty phenotype, described by *Fried et al* in 2001, defines frailty as the presence of three or more out of five features: low hand-grip strength, unintentional weight loss, low physical activity, exhaustion, and slow walking pace.(11) The presence of one or two of these features is classified as

For peer review only - http://bmjopen.bmj.com/site/about/guidelines.xhtml

a pre-frail state. The frailty index, described by Rockwood and Mitnitski in 2007, is based on a cumulative-deficit model of frailty whereby frailty is identified by counting the number of health 'deficits' present in an individual.(12) At least 30 deficits are required to construct a frailty index, all of which must increase in prevalence with age, be associated with poor health, and not saturate too early (i.e. be universally present among older people).(13) Both the frailty phenotype and frailty index have been associated with adverse health outcomes in a range of older populations, however the populations identified as frail by each are different.(14) Since their original description, a wide range of other frailty instruments, as well as adaptations of the frailty index and phenotype, have been developed for both epidemiological studies and for clinical practice.(9, 10)

The relationship between diabetes and frailty is complex. Diabetes is associated with a higher prevalence of frailty.(15-18) Both type 1 and type 2 diabetes lead to microvascular and macrovascular complications which have important physical, cognitive and functional consequences, that may contribute to the development of frailty.(6) Hyperglycaemia is also recognized to directly impact muscle mass and quality, exacerbating age-related sarcopenia and, in turn, physical function.(19) However, the association between frailty and poor functional outcomes in people with diabetes is only partially explained by direct complications of diabetes.(17, 20)

The importance of frailty in the context of diabetes is increasingly recognised in clinical guidelines.(7) Specifically, higher HbA1c targets are recommended in the context of frailty, in part due to the increased risks associated with hypoglycaemia.(21) Despite this, up to 40% of older people with diabetes may be over-treated (with HbA1c <7%).(22, 23) Conversely, poor glycaemic control and associated vascular complications risk causing, or accelerating the progression of, frailty.(24)

One recent meta-analysis demonstrated a consistent relationship between frailty and mortality, hospitalisation, and cardiovascular events in the context of diabetes.(25) We are not aware of any systematic review to assess the prevalence of frailty in diabetes, or to consider a broader range of outcomes relevant to the management of diabetes. Given the risks of both over- and under-

For peer review only - http://bmjopen.bmj.com/site/about/guidelines.xhtml

treatment of diabetes in the context of frailty, understanding the range of potential associations is required to inform clinical decisions and to underpin future research.

To enhance understanding of the implications and management of diabetes within an ageing population, it is important to fully describe the association between diabetes and frailty. Given the risks of both over- and under-treatment of diabetes in the context of frailty, it is important to understand the associations between frailty and a range of potential outcomes in diabetes. This includes generic outcomes such as mortality and hospitalisation and disability and disease specific outcomes such as retinopathy, neuropathy, and hypoglycaemic events. An understanding of the range and complexity of these associations is required to inform clinical decisions around treatment priorities and to underpin future research. This includes quantifying the prevalence of frailty in people with diabetes, and the impact that different frailty definitions might have on this prevalence. This manuscript describes the protocol of a systematic review aiming to synthesise existing evidence relating to these questions.

For peer review only

Aims

The systematic review will aim to:

- Identify which frailty measures have been used to assess frailty in people with diabetes (any type, including mixed/unspecified)
- Quantify the prevalence of frailty among people with diabetes
- Describe the association between frailty and both generic (e.g. mortality) and disease specific (e.g. hypoglycaemia) clinical outcomes in the context of diabetes

For peer review only - http://bmjopen.bmj.com/site/about/guidelines.xhtml

Methods and analysis

This protocol is registered with PROSPERO (CRD42020163109). The review will be conducted and reported according to the Preferred Reporting Items for Systematic Reviews and Meta-Analyses (PRISMA) statement.(26) Where a meta-analysis is undertaken, we will report findings according to the Meta-analyses Of Observational Studies in Epidemiology checklist.

Eligibility criteria for inclusion

The eligibility criteria for this review are summarized in table 1 and explained in more detail below.

Table 1. Inclusion Criteria

PECOS component	Description
Population	Adults (≥ 18 years old)<br>Diabetes (any type, including mixed or unspecified)
Exposure	Frailty as assessed by any frailty measure
Comparator	People with diabetes not classified as frail
Outcomes	Generic:<br>• Mortality<br>• Major Adverse Cardiovascular Events<br>• Hospital admission<br>• Admission to long-term care facility<br>• Falls<br>• Number of clinic attendances<br>• Quality of life<br>• Disability/functional status<br>Diabetes specific:

For peer review only

For peer review only - http://bmjopen.bmj.com/site/about/guidelines.xhtml

	• HbA1c (cross sectional association, or longitudinal)<br>• Glycaemic variability<br>• Hypoglycaemic episodes<br>• Diabetic retinopathy (cross sectional association, or longitudinal)<br>• Diabetic nephropathy (cross sectional association, or longitudinal)<br>○ Include development of end-stage renal disease<br>• Diabetic foot complications (cross sectional association, or longitudinal)<br>• Treatment burden (e.g. Diabetic Treatment Burden Questionnaire)
Settings	Community (including care home/nursing home)<br>Outpatient clinic<br>Inpatient
Study design	Cross sectional or longitudinal<br>Cohort
Other exclusions	Conference abstracts, letters, review articles, intervention studies, Grey literature

Population

We will include studies analysing data from people with any form of diabetes.

While frailty is a state associated with increasing age, there is evidence that frailty is identifiable in relatively younger people, particularly in certain contexts such as multimorbidity (2 or more co-existing long-term conditions) or in areas of high socioeconomic deprivation. We will therefore include studies of adults of any age (≥18 years). However, we anticipate that most studies will focus predominantly on 'older' populations.

For peer review only

For peer review only - http://bmjopen.bmj.com/site/about/guidelines.xhtml

From an initial scoping of the literature, it is likely that many studies describing frailty in population-based studies measure unspecified 'diabetes' rather than explicitly type 1 or type 2 diabetes. We will therefore include any study which includes people with any type of diabetes (including type 1, type 2 diabetes, secondary or monogenic diabetes, or people with unspecified diabetes). Given that frailty is a state associated with older age, and that type 2 diabetes is both more prevalent than type 1 diabetes and becomes more prevalent with age, it is likely that most (but not all) people with diabetes in the relevant populations will have type 2 diabetes. Studies of type 1 diabetes, type 2 diabetes and those of unspecified diabetes will be considered separately in any subsequent analysis.

We will include studies focusing purely on people with diabetes, or population-based studies that report results for people with diabetes separately.

Exposure

The 'exposure' of interest is frailty. Many epidemiological measures and clinical tools have been developed to identify frailty for research or clinical practice.(10)

To be eligible for inclusion, a study must use a measure which explicitly seeks to quantify frailty. We will include measures developed primarily as epidemiological tools (e.g. the frailty phenotype frailty index).(11, 12) We will also include measures designed primarily for clinical practice (e.g. the Clinical Frailty Scale).(27)

Studies focusing solely on comorbidity (i.e. no additional measures to identify 'frailty') will be excluded unless these are explicitly operationalised as a 'frailty index'. In this case studies would generally be expected to include additional deficits (such as symptoms, functional limitations, laboratory measures etc.). Studies which use a single parameter as a proxy for frailty (e.g. grip strength alone, self-rated health) will be excluded.

For peer review only

For peer review only - http://bmjopen.bmj.com/site/about/guidelines.xhtml

Comparator

Studies that report the prevalence of frailty will be eligible for inclusion if they report the prevalence of frailty in diabetes only. Studies should report the number or proportion of participants with and without frailty (or with varying degrees of frailty, depending on the measure used).

For assessing the association between frailty and clinical outcomes in the context of diabetes, studies should report the association between frailty and the outcome of interest. This may be reported either as the association with the presence or absence of frailty (in the case of a binary or categorical measure) or the association between the degree of frailty and the outcome (in the case of a continuous or ordinal measure of frailty).

Outcomes

Outcomes of interest are summarized in table 1. We will include studies assessing any of these outcomes as long as the association is specifically quantified in people with diabetes and frailty.

Setting

We will include studies of community-dwelling patients, outpatient populations or hospital inpatients.

For the purposes of this review, given the focus on frailty, people living in long-term care facilities (e.g. care-homes, nursing-homes) will be considered to be 'community-dwelling'. Therefore, any study including, or specifically recruiting, nursing home residents will be eligible for inclusion.

For peer review only

191 ## Identification of studies

192 ### Electronic searches

193 Medline, Embase, and Web of Science (Core collection) databases will be search using a combination
194 of Medical Subject Headings (MeSH) and keyword searches (Supplementary file 1). The terms used
195 for the medline search are shown in table 2. These terms will be adapted for the other databases.
196 Searches will be from 2000 to November 2019. The year 2000 was chosen as the start date as the
197 first seminal paper operationalising the concept of frailty in an epidemiological study was published
198 in 2001. Articles published prior to this date are therefore unlikely to be relevant. No language
199 restriction will be applied to the search, but only English language articles will be included at the
200 screening level. This language restriction is a pragmatic decision, however we acknowledge that this
201 may lead to a language bias in the results, potentially excluding relevant studies published in other
202 languages.

Table 2: Medline Search
1. Exp Frailty/<br>2. Exp Frail Elderly/<br>3. Frail*.tw<br>4. 1 or 2 or 3<br>5. Exp Diabetes Mellitus<br>6. Diabet*.tw<br>7. (IDDM or NIDDM or MODY or T1DM, or T2DM or T1D or T2D).tw<br>8. (non insulin* depend* or non insulin depend* or non insulin?depend* or non insulin ?depend).tw<br>9. (insulin* depend* or insulin ?depend*).tw<br>10. 5 or 6 or 7 or 8 or 9

For peer review only - http://bmjopen.bmj.com/site/about/guidelines.xhtml

For peer review only

11. Exp Diabetes Insipidus/
12. Diabet* insipidus.tw
13. 11 or 12
14. 10 not 13
15. 4 and 14

Identifying additional articles

Electronic searches will be supplemented by hand searching reference lists of relevant articles. A citation search of all relevant articles will also be carried out using the Web of Science citation search tool.

Data collection and analysis

Selection of studies

Two reviewers, working independently, will screen all titles and abstracts of records identified in the database searches. PECOS criteria outlined above will be used to determine eligibility. Where there is disagreement, studies will be retained for full-text screening.

Full texts of all potentially eligible studies will be screened independently by two reviewers. Disagreements about eligibility will be resolved by consensus, involving a third reviewer where necessary.

Data extraction

A standard data extraction form will be designed and piloted before being applied to each of the included studies. Extracted data will include:

Study details

For peer review only

For peer review only - http://bmjopen.bmj.com/site/about/guidelines.xhtml

- Author
- Year
- Location
- Setting (community, outpatient, residential care)
- Method of recruitment (e.g. random sample, postal invitation, consecutive patients)
- Method of assessment (face-to-face, survey, linkage to healthcare records)

Population

- Age
- Sex
- Ethnicity
- Socioeconomic status
- Comorbidities
- Medications
- Social circumstances (e.g. living independently, requiring carers, family support etc)
- Smoking status
- Physical activity

Diabetes details

- Type of diabetes
- Method of confirmation (self-report, medical records, clinical assessment)
- Measure of control (e.g. HbA1c)
- Medication (e.g. proportion taking insulin, oral antidiabetics etc.)
- Presence and severity of complications (e.g. retinopathy, nephropathy, neuropathy, ulceration, Charcot arthropathy)

Frailty definition

For peer review only

For peer review only - http://bmjopen.bmj.com/site/about/guidelines.xhtml

- Frailty measure used
- Definitions for each component of the frailty measure (e.g. cut-points used for continuous measures, method of assessment (questionnaire, interview etc.))

Frailty prevalence

Outcomes (generic):

- Mortality
- Major Adverse Cardiovascular Events
- Hospital admission
- Admission to long-term care facility
- Falls
- Number of clinic attendances
- Quality of life
- Disability/functional status

Outcomes (diabetes specific):

- HbA1c (cross sectional association, or longitudinal)
- Glycaemic variability
- Hypoglycaemic episodes
- Diabetic retinopathy (cross sectional association, or longitudinal)
- Diabetic nephropathy (cross sectional association, or longitudinal)
- Diabetic foot complications (cross sectional association, or longitudinal)
- Treatment burden (e.g. Diabetic Treatment Burden Questionnaire)

As we include a wide range of outcomes, it is likely that the way outcomes are assessed will vary depending on the outcome in question. Studies may also assess similar outcomes (e.g. hospital admission) in different ways (e.g. number of admissions over specified follow-up, time to first

For peer review only

For peer review only - http://bmjopen.bmj.com/site/about/guidelines.xhtml

267 admission, presence of absence of admission during follow-up). For the outcomes listed above, we
268 will extract data regardless of the method of assessment. Heterogeneity in the way outcome data
269 were collected will be used to inform the approach to data synthesis (i.e. meta-analysis versus
270 narrative synthesis). For each outcome reported we will record

271 • The method of outcome assessment (e.g. linkage to healthcare records, face-to-face
272 assessment, questionnaire etc.)
273 • Method of analysis (e.g. time-to-event, mean difference etc.)
274 • The association between frailty and the outcome (e.g. prevalence, odds ratio, hazard ratio
275 etc.)
276 • Adjustment for any potential confounders
277 • Length of follow-up over which the outcome was assessed
278 • Method of analysis of competing risks when assessing each outcome.

279 Where available, we will also extract data on both relative (e.g. hazard ratios) and absolute (e.g.
280 events per 1000 people) associations with outcomes.

281 Assessment of methodological quality

282 The Newcastle-Ottawa scale will be used to assess the risk of bias for each study (Supplementary file
283 2).(28) This scale is widely used for the assessment of observational studies, and has frequently been
284 adapted to the context of specific systematic reviews. We have adapted the criteria in order to be
285 explicit about how the 'exposure assessment' related to frailty: specifically, awarding one point for
286 the use of a validated frailty assessment measure. For cross-sectional studies, only the first 5
287 elements of the scale were relevant to quality assessment (the remainder concerning the
288 longitudinal assessment of outcomes). We will use this subsection of the Newcastle-Ottawa scale to
289 assess the quality of cross-sectional studies to allow direct comparability with the baseline
290 assessments of longitudinal studies (from which we will also extract data on frailty prevalence). In

For peer review only

assessing the comparability of frail/non-frail groups, age will be taken as the most important factor for which studies should account.

Data synthesis

The appropriate method of data synthesis will be determined after assessment of the heterogeneity of the included studies, in terms of population selection and demographics, frailty definition, and method of outcome assessment.

With regards to the prevalence of frailty, different frailty measures will be considered separately (i.e. we will not perform a meta-analysis of frailty prevalence measured using different scales). We will also consider community studies separately from studies focussing on outpatient clinic populations (as these may represent people with more severe diabetes), inpatients or people living in residential care. We will also assess the inclusion criteria and demographics of the sample population, with particular attention to age (as frailty is strongly associated with age) and sex (as women tend to have a higher prevalence of frailty than men) to determine the most appropriate method of synthesis. Where samples have been drawn from populations with a markedly different age/sex structure, a pooled estimate of the mean prevalence of frailty across these studies is unlikely to be a meaningful summary. Similarly, other inclusion criteria used by the individual studies (such as excluding 'institutionalised' people, people with cognitive impairment, of people with impaired mobility unable to attend an assessment) may disproportionately impact on the estimation of frailty prevalence. The appropriateness, or otherwise, of a meta-analysis of frailty prevalence will be judged only after examination of these aspects of the included studies.

For the assessment of outcomes, the approach to synthesis will also be judged based on heterogeneity of the method of outcome assessment and the analytic approach. As above, different frailty measures will be considered separately.

For peer review only

If appropriate, we will combine these in a random effects meta-analysis (anticipating heterogeneity in the true association). As well as a pooled estimate and 95% confidence intervals, we will also calculate the prediction interval to assess the range of plausible estimates from the observed data. Heterogeneity will be quantified using the I^2 statistic. Where heterogeneity is present, we will attempt to explore potential sources of heterogeneity using subgroup analyses (e.g. by method of determining frailty, age of sample population, method of outcome assessment). By doing so, we propose to explore factors that may influence the estimates reported in observational studies in the presence of heterogeneity, rather than provide a definitive single estimate.(29) We will use funnel plots to assess for potential publication bias.

Only those studies that are judged to be sufficiently comparable will be included in meta-analyses. For outcomes where there are too few studies, or the included studies are too heterogenous to permit a meaningful meta-analysis (for example, in terms of outcome definition or method of assessing frailty), we will perform a narrative synthesis of the study findings. This will report the methods used to identify frailty along with the prevalence and association with outcomes, to explore the impact of the method of assessment on the observed relationship. This will be reported alongside detail of the recruitment strategy, age profile, and characteristics of each sample included.

Patient and public involvement

No patients were involved in the development of this review.

For peer review only

For peer review only - http://bmjopen.bmj.com/site/about/guidelines.xhtml

Ethics and dissemination

This systematic review will provide an overview of the prevalence of frailty in diabetes, and the relationship between frailty and adverse health outcomes in people with diabetes.

As the prevalence of both frailty and diabetes increase, it will become increasingly important for clinical guidelines for the treatment of diabetes to explicitly consider the needs of people living with frailty.(7) Quantifying the prevalence of frailty in diabetes will allow the scale of this challenge to be better appreciated. By including any reported definition of frailty within our inclusion criteria, this review will demonstrate which of the wide range of frailty instruments and measures have been used to study frailty in diabetes. It will also be possible to compare if and how prevalence and association with outcomes differs depending on the frailty definition used.

Given the likely heterogeneity in frailty definitions, as well as inherent differences in the populations studied, it may not be possible to undertake a meta-analysis of the findings of this review. If this is the case, we propose to conduct a detailed narrative synthesis, systematically describing and synthesizing details of the populations under study as well as the details of frailty definitions used.

We also propose to search for and extract data for a wide range of clinical outcomes. Given the multidimentional nature of frailty,(8) and the vulnerability to decompensation that is inherent to any frailty definition,(9) it is likely that frailty will be associated with a range of adverse outcomes. The challenge in translating these associations into meaningful recommendations is understanding the balance of these risks, and how they might inform clinical decisions and recommendations. The balance of risks in diabetes, and treatment priorities, may differ depending on the degree of frailty experienced by an individual. The associations may also differ in their nature or magnitude depending on the method used to identify frailty. This review will aim to provide an overview of what is known about the relationship between frailty and both generic and disease specific

For peer review only - http://bmjopen.bmj.com/site/about/guidelines.xhtml

outcomes. This is likely to inform priorities for future research into the consequences of frailty in diabetes.

As this project is a systematic review, ethical approval is not required. Patients or the public were not involved in the development of this protocol.

For peer review only

For peer review only - http://bmjopen.bmj.com/site/about/guidelines.xhtml

Author contributions

All authors (PH, IF, NC, EB, JL, DM and FM) contributed to the conception and design of the proposed study. PH, DM and FM developed the data sources and search strategy. PH, IF, NC, DM and FM refined the inclusion criteria. PH, IF, NC, DM and FM developed the data extraction template which was piloted by PH, IF and NC. PH and IF wrote the first draft. All authors critically reviewed this and subsequent drafts. All authors approved the final version of the manuscript for submission. FM is the guarantor of the review. All authors accept accountability for the accuracy of the protocol.

Funding statement

Dr Peter Hanlon was funded by a Medical Research Council Clinical Research Training Fellowship (Grant reference MR/S021949/1) entitled “Understanding prevalence and impact of frailty in chronic disease and implications for clinical management. The funder had no role in protocol development.

Competing interests statement

The authors declare no competing interests.

For peer review only

References

1. Moini J. Epidemiology of Diabetes: Elsevier; 2019.
2. Ozougwu J, Obimba K, Belonwu C, Unakalamba C. The pathogenesis and pathophysiology of type 1 and type 2 diabetes mellitus. Journal of physiology and pathophysiology. 2013;4(4):46-57.
3. Pambianco G, Costacou T, Ellis D, Becker DJ, Klein R, Orchard TJ. The 30-year natural history of type 1 diabetes complications: the Pittsburgh Epidemiology of Diabetes Complications Study experience. Diabetes. 2006;55(5):1463-9.
4. Shaw JE, Sicree RA, Zimmet PZ. Global estimates of the prevalence of diabetes for 2010 and 2030. Diabetes research and clinical practice. 2010;87(1):4-14.
5. United Nations. World Population Ageing [Report] 2015 [Available from: https://www.un.org/en/development/desa/population/publications/pdf/ageing/WPA2015_Report.pdf.
6. Sinclair AJ, Rodriguez-Manas L. Diabetes and Frailty: Two Converging Conditions? Canadian journal of diabetes. 2016;40(1):77-83.
7. Sinclair AJ, Abdelhafiz A, Dunning T, Izquierdo M, Manas LR, Bourdel-Marchasson I, et al. An international position statement on the management of frailty in diabetes mellitus: summary of recommendations 2017. The Journal of frailty & aging. 2018;7(1):10-20.
8. Clegg A, Young J, Iliffe S, Rikkert MO, Rockwood K. Frailty in elderly people. The Lancet. 2013;381(9868):752-62.
9. Hoogendijk EO, Afilalo J, Ensrud KE, Kowal P, Onder G, Fried LP. Frailty: implications for clinical practice and public health. The Lancet. 2019;394(10206):1365-75.
10. Bouillon K, Kivimaki M, Hamer M, Sabia S, Fransson EI, Singh-Manoux A, et al. Measures of frailty in population-based studies: an overview. BMC Geriatrics. 2013;13(1):64.
11. Fried LP, Tangen CM, Walston J, Newman AB, Hirsch C, Gottdiener J, et al. Frailty in Older Adults: Evidence for a Phenotype. The Journals of Gerontology: Series A. 2001;56(3):M146-M57.
12. Rockwood K, Mitnitski A. Frailty in Relation to the Accumulation of Deficits. The Journals of Gerontology: Series A. 2007;62(7):722-7.
13. Searle SD, Mitnitski A, Gahbauer EA, Gill TM, Rockwood K. A standard procedure for creating a frailty index. BMC Geriatrics. 2008;8(1):24.
14. Santos-Eggimann B, Sirven N. Screening for frailty: older populations and older individuals. Public health reviews. 2016;37(1):7.
15. Kotsani M, Chatziadamidou T, Economides D, Benetos A. Higher prevalence and earlier appearance of geriatric phenotypes in old adults with type 2 diabetes mellitus. Diabetes Research and Clinical Practice. 2018;135:206-17.
16. Thein FS, Li Y, Nyunt MSZ, Gao Q, Wee SL, Ng TP. Physical frailty and cognitive impairment is associated with diabetes and adversely impact functional status and mortality. Postgraduate medicine. 2018;130(6):561-7.
17. Castro-Rodriguez M, Carnicero JA, Garcia-Garcia FJ, Walter S, Morley JE, Rodriguez-Artalejo F, et al. Frailty as a Major Factor in the Increased Risk of Death and Disability in Older People With Diabetes. Journal of the American Medical Directors Association. 2016;17(10):949-55.
18. Hanlon P, Hannigan L, Fischbacker C, Welton N, Dias S, Mair F, et al. Representation of people with comorbidity and multimorbidity in clinical trials of novel drug therapies; an individual-level participant data analysis. BMC Medicine (in press). 2019.
19. Yoon JW, Ha Y-C, Kim KM, Moon JH, Choi SH, Lim S, et al. Hyperglycemia Is Associated with Impaired Muscle Quality in Older Men with Diabetes: The Korean Longitudinal Study on Health and Aging. Diabetes Metab J. 2016;40(2):140-6.
20. Maggi S, Noale M, Gallina P, Marzari C, Bianchi D, Limongi F, et al. Physical disability among older Italians with diabetes. The ILSA Study. Diabetologia. 2004;47(11):1957-62.

For peer review only - http://bmjopen.bmj.com/site/about/guidelines.xhtml

21. Schernthaner G, Schernthaner-Reiter MH. Diabetes in the older patient: heterogeneity requires individualisation of therapeutic strategies. Diabetologia. 2018;61(7):1503-16.

22. Formiga F, Franch-Nadal J, Rodriguez L, Ávila L, Fuster E. Inadequate glycaemic control and therapeutic management of adults over 65 years old with type 2 diabetes mellitus in Spain. The journal of nutrition, health & aging. 2017;21(10):1365-70.

23. Braun AK, Kubiak T, Kuntsche J, Meier-Höfig M, Müller UA, Feucht I, et al. SGS: a structured treatment and teaching programme for older patients with diabetes mellitus—a prospective randomised controlled multi-centre trial. Age and ageing. 2009;38(4):390-6.

24. Quartuccio M, Buta B, Kalyani RR. Comparative effectiveness for glycemic control in older adults with diabetes. Current geriatrics reports. 2017;6(3):175-86.

25. Ida S, Kaneko R, Imataka K, Murata K. Relationship between frailty and mortality, hospitalization, and cardiovascular diseases in diabetes: a systematic review and meta-analysis. Cardiovascular Diabetology. 2019;18(1):81.

26. Moher D, Liberati A, Tetzlaff J, Altman DG. Preferred reporting items for systematic reviews and meta-analyses: the PRISMA statement. BMJ. 2009;339:b2535.

27. Rockwood K, Song X, MacKnight C, Bergman H, Hogan DB, McDowell I, et al. A global clinical measure of fitness and frailty in elderly people. Canadian Medical Association Journal. 2005;173(5):489.

28. Stang A. Critical evaluation of the Newcastle-Ottawa scale for the assessment of the quality of nonrandomized studies in meta-analyses. European Journal of Epidemiology. 2010;25(9):603-5.

29. Mueller M, D'Addario M, Egger M, Cevallos M, Dekkers O, Mugglin C, et al. Methods to systematically review and meta-analyse observational studies: a systematic scoping review of recommendations. BMC Medical Research Methodology. 2018;18(1):44.

For peer review only

For peer review only - http://bmjopen.bmj.com/site/about/guidelines.xhtml

Medline Search Strategy

Search Terms

1. Exp Frailty/
2. Exp Frail Elderly/
3. Frail*.tw
4. 1 or 2 or 3
5. Exp Diabetes Mellitus
6. Diabet*.tw
7. (IDDM or NIDDM or MODY or T1DM, or T2DM or T1D or T2D).tw
8. (non insulin* depend* or non insulin depend* or non insulin?depend* or non insulin ?depend).tw
9. (insulin* depend* or insulin ?depend*).tw
10. 5 or 6 or 7 or 8 or 9
11. Exp Diabetes Insipidus/
12. Diabet* insipidus.tw
13. 11 or 12
14. 10 not 13
15. 4 and 14

Language restriction

None applied to search (non-English language studies excluded at screening stage)

Years searched

2001-November 2019

For peer review only

The Newcastle-Ottawa Scale

Adaptation for studies assessing the prevalence and impact of frailty in diabetes

1 – Representativeness of the exposed (i.e. frail) cohort

a) Truly representative (one star)

b) Somewhat representative (one star)

c) Selected group

d) No description of the derivation of the cohort

2 – Selection of the non-exposed (i.e. non-frail) cohort

a) Drawn from the same community as the exposed cohort (one star)

b) Drawn from a different source

c) No description of the derivation of the non-exposed cohort

3 – Ascertainment of exposure

a) Validated measurement tool for frailty (two stars)

b) Non-validated measurement tool, but the tool is available or described (one star)

c) No description of measurement tool

4 – Non-respondents

a) Comparability between respondents and non-respondents' characteristics is established, and the response rate is satisfactory (one star)
b) The response rate is unsatisfactory, or the comparability between respondents and non-respondents is unsatisfactory

c) No description of the response rate of the characteristics of the responders and non-responders

5 – Demonstration that outcome of interest was not present at the start of the study

a) Yes (one star)

b) No

Comparability:

1 – Comparability of the cohorts on the basis of the design or analysis being controlled for confounders

a) The study controls for age and sex (one star)

b) The study controls for other factors (one star)

c) Cohorts are not comparable on the basis of the design or analysis controlled for confounders

For peer review only

Outcomes:

1 – Assessment of outcomes

a) Independent assessment (one star)

b) Record linkage (one star)

c) Self-report

d) No description

e) Other

2 – Follow-up long enough for outcomes to occur

a) Yes (one star)

b) No

3 – Adequacy of follow-up of cohorts

a) Complete follow-up: all subjects accounted for (one star)

b) Subjects lost to follow-up unlikely to introduce bias – number lost less than or equal to 20% or description of those lost suggested no different from those followed (one star)

c) Follow-up rate less than 80% and no description of those lost

d) No statement

For peer review only

For peer review only

Reporting checklist for protocol of a systematic review.

Based on the PRISMA-P guidelines.

Instructions to authors

Complete this checklist by entering the page numbers from your manuscript where readers will find each of the items listed below.

Your article may not currently address all the items on the checklist. Please modify your text to include the missing information. If you are certain that an item does not apply, please write "n/a" and provide a short explanation.

Upload your completed checklist as an extra file when you submit to a journal.

In your methods section, say that you used the PRISMA-Preporting guidelines, and cite them as:

Moher D, Shamseer L, Clarke M, Ghersi D, Liberati A, Petticrew M, Shekelle P, Stewart LA. Preferred Reporting Items for Systematic Review and Meta-Analysis Protocols (PRISMA-P) 2015 statement. Syst Rev. 2015;4(1):1.

For peer review only

		Reporting Item	Page Number
**Title**			
Identification	#1a	Identify the report as a protocol of a systematic review	1
Update	#1b	If the protocol is for an update of a previous systematic review, identify as such	n/a

**Registration**			
	#2	If registered, provide the name of the registry (such as PROSPERO) and registration number	3
**Authors**			
Contact	#3a	Provide name, institutional affiliation, e-mail address of all protocol authors; provide physical mailing address of corresponding author	1
Contribution	#3b	Describe contributions of protocol authors and identify the guarantor of the review	18
**Amendments**			
	#4	If the protocol represents an amendment of a previously completed or published protocol, identify as such and list changes; otherwise, state plan for documenting important protocol amendments	n/a
**Support**			
Sources	#5a	Indicate sources of financial or other support for the review	18
Sponsor	#5b	Provide name for the review funder and / or sponsor	18
Role of sponsor or funder	#5c	Describe roles of funder(s), sponsor(s), and / or institution(s), if any, in developing the protocol	18
**Introduction**			

For peer review only

Rationale	#6	Describe the rationale for the review in the context of what is already known	4,5
Objectives	#7	Provide an explicit statement of the question(s) the review will address with reference to participants, interventions, comparators, and outcomes (PICO)	6
**Methods**			
Eligibility criteria	#8	Specify the study characteristics (such as PICO, study design, setting, time frame) and report characteristics (such as years considered, language, publication status) to be used as criteria for eligibility for the review	7-10
Information sources	#9	Describe all intended information sources (such as electronic databases, contact with study authors, trial registers or other grey literature sources) with planned dates of coverage	10
Search strategy	#10	Present draft of search strategy to be used for at least one electronic database, including planned limits, such that it could be repeated	10-11
Study records - data management	#11a	Describe the mechanism(s) that will be used to manage records and data throughout the review	11
Study records - selection process	#11b	State the process that will be used for selecting studies (such as two independent reviewers) through each phase of the review (that is, screening, eligibility and inclusion in meta-analysis)	11-12

For peer review only

Study records - data collection process	#11c	Describe planned method of extracting data from reports (such as piloting forms, done independently, in duplicate), any processes for obtaining and confirming data from investigators	12-13
Data items	#12	List and define all variables for which data will be sought (such as PICO items, funding sources), any pre-planned data assumptions and simplifications	12-13
Outcomes and prioritization	#13	List and define all outcomes for which data will be sought, including prioritization of main and additional outcomes, with rationale	13-14
Risk of bias in individual studies	#14	Describe anticipated methods for assessing risk of bias of individual studies, including whether this will be done at the outcome or study level, or both; state how this information will be used in data synthesis	14
Data synthesis	#15a	Describe criteria under which study data will be quantitatively synthesised	14-15
Data synthesis	#15b	If data are appropriate for quantitative synthesis, describe planned summary measures, methods of handling data and methods of combining data from studies, including any planned exploration of consistency (such as I2, Kendall's τ)	14-15
Data synthesis	#15c	Describe any proposed additional analyses (such as sensitivity or subgroup analyses, meta-regression)	n/a

For peer review only

Data synthesis	#15d	If quantitative synthesis is not appropriate, describe the type of summary planned	15
Meta-bias(es)	#16	Specify any planned assessment of meta-bias(es) (such as publication bias across studies, selective reporting within studies)	n/a
Confidence in cumulative evidence	#17	Describe how the strength of the body of evidence will be assessed (such as GRADE)	n/a

None The PRISMA-P checklist is distributed under the terms of the Creative Commons Attribution License CC-BY 4.0. This checklist can be completed online using https://www.goodreports.org/, a tool made by the EQUATOR Network in collaboration with Penelope.ai

For peer review only
